# The Global Atmosphere‐aerosol Model ICON‐A‐HAM2.3–Initial Model Evaluation and Effects of Radiation Balance Tuning on Aerosol Optical Thickness

**DOI:** 10.1029/2021MS002699

**Published:** 2022-04-02

**Authors:** M. Salzmann, S. Ferrachat, C. Tully, S. Münch, D. Watson‐Parris, D. Neubauer, C. Siegenthaler‐Le Drian, S. Rast, B. Heinold, T. Crueger, R. Brokopf, J. Mülmenstädt, J. Quaas, H. Wan, K. Zhang, U. Lohmann, P. Stier, I. Tegen

**Affiliations:** ^1^ Institute for Meteorology Universität Leipzig Leipzig Germany; ^2^ Institute of Atmospheric and Climate Science ETH Zürich Zürich Switzerland; ^3^ Atmospheric, Oceanic and Planetary Physics Department of Physics University of Oxford Oxford UK; ^4^ Center for Climate Systems Modeling ETH Zürich Zürich Switzerland; ^5^ Max Planck Institute for Meteorology Hamburg Germany; ^6^ Leibniz Institute for Tropospheric Research Leipzig Germany; ^7^ Now at Pacific Northwest National Laboratory Richland WA USA; ^8^ Pacific Northwest National Laboratory Richland WA USA

**Keywords:** aerosol, modeling

## Abstract

The Hamburg Aerosol Module version 2.3 (HAM2.3) from the ECHAM6.3‐HAM2.3 global atmosphere‐aerosol model is coupled to the recently developed icosahedral nonhydrostatic ICON‐A (icon‐aes‐1.3.00) global atmosphere model to yield the new ICON‐A‐HAM2.3 atmosphere‐aerosol model. The ICON‐A and ECHAM6.3 host models use different dynamical cores, parameterizations of vertical mixing due to sub‐grid scale turbulence, and parameter settings for radiation balance tuning. Here, we study the role of the different host models for simulated aerosol optical thickness (AOT) and evaluate impacts of using HAM2.3 and the ECHAM6‐HAM2.3 two‐moment cloud microphysics scheme on several meteorological variables. Sensitivity runs show that a positive AOT bias over the subtropical oceans is remedied in ICON‐A‐HAM2.3 because of a different default setting of a parameter in the moist convection parameterization of the host models. The global mean AOT is biased low compared to MODIS satellite instrument retrievals in ICON‐A‐HAM2.3 and ECHAM6.3‐HAM2.3, but the bias is larger in ICON‐A‐HAM2.3 because negative AOT biases over the Amazon, the African rain forest, and the northern Indian Ocean are no longer compensated by high biases over the sub‐tropical oceans. ICON‐A‐HAM2.3 shows a moderate improvement with respect to AOT observations at AERONET sites. A multivariable bias score combining biases of several meteorological variables into a single number is larger in ICON‐A‐HAM2.3 compared to standard ICON‐A and standard ECHAM6.3. In the tropics, this multivariable bias is of similar magnitude in ICON‐A‐HAM2.3 and in ECHAM6.3‐HAM2.3. In the extra‐tropics, a smaller multivariable bias is found for ICON‐A‐HAM2.3 than for ECHAM6.3‐HAM2.3.

## Key Points

- The new ICON‐A‐Hamburg Aerosol Module version 2.3 (HAM2.3) global atmosphere‐aerosol model is introduced
- The sensitivity of simulated aerosol optical thickness (AOT) to using two different host models but the same aerosol module is investigated
- A positive AOT bias over subtropical oceans is remedied in ICON‐A‐HAM2.3 because of a different parameter setting in the host model

## 1 Introduction

Global atmosphere models coupled with dedicated aerosol modules have frequently been used to estimate the global mean radiative forcing due to aerosol‐radiation and aerosol‐cloud interactions (ERFari + aci, e.g., Boucher et al., 2013). The initial development of such models typically involves coupling an aerosol module to an existing global atmosphere model (e.g., Stier et al., 2005). Subsequently, the aerosol modules as well as the host models are further developed and the results of this development are documented. Usually, this involves an evaluation of how the modifications to the aerosol module and/or the host model affect selected model results in comparison to available observations (e.g., Neubauer et al., 2019; Tegen et al., 2019).

For global atmosphere‐aerosol models, the evaluation of simulated aerosol optical thickness (AOT) has traditionally focused on changes to the aerosol module. However, it may be expected that the outcomes of model simulations with coupled atmosphere‐aerosol models are not only affected by the aerosol module, but also by the choice of the host model. Host models usually differ not only with regard to their dynamical cores but also with regard to their physics parameterization. Understanding the effect of the host model for example, on simulated AOT is further complicated by the need for radiation balance tuning in the host model, typically via adjusting values of poorly constrained parameters in the physics parameterization schemes (e.g., Mauritsen et al., 2012).

The net radiation at the top of the atmosphere is a small difference of two large terms, namely the net absorbed solar radiation and the outgoing terrestrial (or long‐wave) radiation. Therefore, replacing or modifying a physics parameterization or applying changes to the dynamical core will typically require re‐tuning the host model for radiation balance. Thus, in switching to a new host model with a different dynamical and a similar physics package, not only the changes to the dynamical core and the physics package, but also radiation balance tuning, involving parameter settings in the host model physics parameterizations, can in principle affect model results such as simulated AOTs. Furthermore, once an aerosol module is coupled to a new host model, additional radiation balance tuning is required.

Unfortunately, such steps are often not documented in sufficient detail, and it can therefore be difficult to understand how much they can actually affect the results, for example, with respect to simulated AOT, at least in some models. Here, we address the question of how much radiation balance tuning affects AOTs in coupling an aerosol module to a different host model. Because coupling an aerosol module and a two‐moment microphysics parameterization to a host model affects meteorology, an initial evaluation of meteorological output can also be considered part of documenting the coupling process.

In order to understand whether for example, a difference of the simulated AOT between two models with different dynamical cores and similar physics packages is indeed caused by the different dynamical cores or the change in the physics package or instead can be attributed to the radiation balance tuning in the host model, one can use a method involving dedicated sensitivity experiments in which tuning parameters from the original host model are applied in the new host model. If changing a particular radiation balance tuning parameter back to the value that was applied in the original host model results in qualitatively very similar features of the simulated AOT compared to the original coupled model, then a change in the AOT results can be attributed to a change in that particular parameter setting and it becomes extremely unlikely that this particular change is instead caused by switching to a different dynamical core or a change in the physics package.

Because this process of attributing changes in the results to changes in the model formulation can be rather elaborate and may require a large number of sensitivity runs, at least initially, it is practical to focus on a few key differences, such as a long‐standing AOT bias that has largely disappeared in a newly developed model, especially if after coupling the aerosol module to the new host model, it is not a‐priori clear why this bias has largely disappeared.

The advantage of using such sensitivity studies is that one avoids misattributing changes in a variable such as AOT to a different dynamical core or a change in the physics formulation when instead the change is caused by a change in the radiation balance tuning. Because host model development should always be expected to involve radiation balance tuning, this kind of detailed study based on numerous sensitivity experiments is a prerequisite to understanding which aspects of a host model affect the simulated AOTs.

Here, we introduce the newly developed ICON‐A‐HAM2.3 atmosphere‐aerosol model. ICON‐A (Giorgetta et al., 2018) is a new atmosphere model, designed as the successor of the well‐established ECHAM6 (Stevens et al., 2013) atmosphere model. ICON‐A‐HAM2.3 is developed based on ECHAM6.3‐HAM2.3. ECHAM‐HAM, originally developed by Stier et al. (2005), has been used in numerous process studies and frequently contributed to model evaluation and intercomparison studies (Tegen et al., 2019).

Both, ICON‐A‐HAM2.3 and ECHAM6.3‐HAM2.3 use prognostic variables to describe aerosol. Aerosol precursors and aerosols are emitted, transported, transformed, and removed. The optical properties of the prognostic aerosol are used in the radiation parameterization to account for interactions of aerosol and radiation. Furthermore, ICON‐A‐HAM2.3 and ECHAM6.3‐HAM2.3 include a two‐moment cloud microphysics scheme with prognostic cloud droplet and cloud ice crystal numbers (Lohmann & Neubauer, 2018; Lohmann et al., 2007), which facilitates the computation of the effective radiative forcing via aerosol‐cloud interactions (ERFaci).

ECHAM6.3‐HAM2.3 is the atmosphere‐aerosol component of the atmosphere‐aerosol‐chemistry model ECHAM6.3‐HAM2.3‐MOZ1.0 (Schultz et al., 2018), which in addition to the HAM2.3 aerosol module (Stier et al., 2005) includes the MOZ1.0 chemistry component. Here, ECHAM6.3‐HAM2.3 is run without a comprehensive chemistry component. Instead, a simple chemistry module that relies on prescribed precomputed oxidant fields is used. However, the so‐called submodel interface (a collection of Fortran subroutines) which is part of the ECHAM6.3‐HAM2.3‐MOZ1.0 infrastructure was used in the coupling. This would in principle facilitate the inclusion of an air chemistry component into ICON‐A‐HAM2.3 in the future.

ICON‐A (icon‐aes‐1.3.00) and ECHAM6.3 share the same physics parameterization package except a change to the parameterization of vertical mixing due to sub‐grid scale turbulence (see Section 2.3), which provides a rather unique setting for investigating the effects of the host model dynamics and different host model tunings on the aerosol simulations. In order to understand how the simulated meteorology in a coupled atmosphere‐aerosol model depends on the host model in our study, we also compare the simulations between host models without aerosol modules and the respective coupled versions.

The following sections briefly describe the ECHAM6.3 and the ICON‐A host models. Section 2.3 provides an overview of the Max Planck Institute physics package, including modifications used in ECHAM6.3‐HAM2.3 and ICON‐A‐HAM2.3. The aerosol module HAM2.3 is described in Section 2.4. The general model setup, parameter settings, and two sets of sensitivity runs to help understand the impacts of radiation balance tunings in ICON‐A‐HAM2.3 and to help understand differences between ECHAM6.3‐HAM2.3 and ICON‐A‐HAM2.3 due to using different host models are described in Sections 2.5 to 2.7. The runs used to compute ERFari + aci and additional sensitivity runs are introduced in Section 2.8. Section 3 introduces observations used for model evaluation. This allows us to evaluate differences between ECHAM6.3‐HAM2.3 and ICON‐A‐HAM2.3 in relation to a set of observations. In Sections 4.1 to 4.6, we first describe and evaluate aspects of the aerosol simulations and then investigate reasons for differences between ECHAM6.3‐HAM2.3 and ICON‐A‐MAM2.3 with respect to AOT observations based on results from sensitivity runs. ERFari + aci is computed in Section 4.7. In Sections 4.8 and 4.9, several aspects of the simulation of meteorological variables are evaluated with observations and compared between ICON‐A‐HAM2.3, ECHAM6.3‐HAM2.3, and the respective host models, which feature different parameter settings and cloud microphysics parameterizations. Numerical noise in both models is discussed in Section 4.10 and Section 5 summarizes the results and highlights several open issues.

## 2 Model Description, Setup, and Sensitivity Runs

### 2.1 The ECHAM6.3 Global Atmosphere Model

The global atmosphere model ECHAM6.3 was developed at the Max Planck Institute for Meteorology (MPI‐M) in Hamburg, Germany. It is based on ECHAM6 (Stevens et al., 2013), but contains several modifications (Mauritsen et al., 2019). ECHAM6.3 is the source of the Max Planck Institute physics package described in Section 2.3.

ECHAM6.3 contains a spectral dynamical core. Spectral coefficients are transformed to variables on a Gaussian grid for evaluating non‐linear products, parameterizations, and tracer transport. Tracer transport is computed using a semi‐Lagrangian advection scheme in flux form based on Lin and Rood (1996). As in many other models, artificial diffusion is added to stabilize the numerical solution (Stevens et al., 2013). In spite of this added diffusion, ECHAM shows a fairly distinct wave pattern for example, west of the Andes over the Pacific Ocean in many of the results. This can be seen for example, in Figure 19 of Stevens et al. (2013) and will be further discussed in Section 4.10.

### 2.2 The ICON‐A Global Atmosphere Model

The ICON‐A (icon‐aes‐1.3.00) global atmosphere model (Giorgetta et al., 2018) is the icosahedral nonhydrostatic (ICON) atmosphere model in a configuration that uses the Max Planck Institute physics package. The ICON model is developed in collaboration between MPI‐M and the German Meteorological Service/Deutscher Wetterdienst (DWD) together with several partner institutes.

The horizontal grid is obtained by projecting an icosahedron onto a sphere which is repeatedly subdivided into smaller spherical triangular cells (Giorgetta et al., 2018; Wan et al., 2013). Because the truncation error of the finite difference divergence operator changes sign between adjacent triangles, artificial diffusion is added to stabilize the numerical solution (Wan et al., 2013). Time integration is performed with a two‐time‐level predictor‐corrector scheme (Zängl et al., 2014). Horizontal tracer transport is computed with the Miura (2007) second order accurate (based on a piecewise linear approximation) upwind‐biased advection scheme with a Zalesak (1979) positive definite flux limiter. Grid‐scale vertical tracer transport is computed with the third‐order accurate piecewise parabolic advection scheme (PPM; Colella & Woodward, 1984).

A main advantage of the ICON‐A dynamical core over the ECHAM6.3 dynamical core is local mass conservation and mass‐consistent tracer transport (Zängl et al., 2014), thus addressing the so‐called mass‐wind inconsistency problem (Jöckel et al., 2001). Another advantage is the absence of a pole problem, which makes this dynamical core particularly well‐suited for investigating processes involving the Arctic and Antarctic. Finally, at very high future model resolutions, the compute time for grid point models such as ICON‐A is expected to scale better with the number of compute nodes on large parallel computers compared to spectral transform models because the transformations in the latter type of models are inherently non‐local, increasing the amount of communication between compute nodes. The transition to very high resolutions is furthermore facilitated by the non‐hydrostatic dynamical core. At the resolutions applied here, this is not yet an issue.

### 2.3 The Max Planck Institute Physics Package

The Max Planck Institute physics package is described in Stevens et al. (2013) with modifications described by Mauritsen et al. (2019) and Giorgetta et al. (2018). Radiative heating and cooling is computed with the PSrad implementation (Pincus & Stevens, 2009) of the rapid radiative transfer model (RRTM; Mlawer et al., 1997) optimized for general circulation models (RRTMG; Iacono et al., 2008; Morcrette et al., 2008). It uses 112 quadrature points in 16 shortwave bands ranging from 820 to 50,000 cm^−1^ and 140 quadrature points in 14 longwave bands in the range from 10 to 3,250 cm^−1^. Cloud overlap is treated using a Monte Carlo Independent Column Approximation (McICA; Pincus et al., 2003) as described by Giorgetta et al. (2018) and Mauritsen et al. (2019). Stratiform cloud cover is diagnosed as a function of relative humidity based on Sundqvist et al. (1989).

Moist convection is parameterized based on the mass‐flux scheme by Tiedtke (1989) with modifications by Nordeng (1994). When activated, the scheme allows for either deep, mid‐level, or shallow convection in the vertical column above a given grid point. The entrainment rates for various types of convection and for downdrafts are prescribed depending on the model configuration (see Section 2.6). Deep convection is assumed to mix readily with the environment due to an organized entrainment and detrainment term and takes precedence over mid‐level and shallow convection (Stevens et al., 2013).

Sub‐grid mountain drag is parameterized following Lott (1999) and nonorographic gravity wave drag is parameterized following Hines (1997). ICON‐A is currently coupled to a simplified version of the land surface model JSBACH (Raddatz et al., 2007) called JSBACH4‐lite (Giorgetta et al., 2018) while ECHAM6.3 is used in combination with the land‐surface model JSBACH3.1.

In ECHAM6.3, vertical transport due to sub‐grid scale turbulence is parameterized based on the turbulent kinetic energy (TKE) scheme by Brinkop and Roeckner (1995). In ICON‐A this scheme was replaced by a total turbulent energy (TTE) scheme based on Mauritsen et al. (2007) and Pithan et al. (2015), which uses different stability functions for the stable boundary layer and a different parameterization of entrainment mixing compared to the scheme by Brinkop and Roeckner (1995). The implementation of the TTE scheme in ICON‐A is described in Giorgetta et al. (2018).

In standard ECHAM6.3 and ICON‐A, cloud liquid and cloud ice mass concentrations in stratiform clouds are computed prognostically while large‐scale stratiform precipitation is treated diagnostically. Stratiform cloud microphysics is parameterized based on Lohmann and Roeckner (1996).

In ICON‐A‐HAM2.3 and ECHAM6.3‐HAM2.3, cloud droplet and ice crystal number concentrations are treated prognostically. Droplet activation is computed by the Abdul‐Razzak and Ghan (2000) parameterization and the modified two‐moment Lohmann et al. (2007) cloud microphysics scheme (Lohmann & Hoose, 2009; Lohmann & Neubauer, 2018; Lohmann et al., 2007) is used. Aerosol concentrations are computed instead of prescribed.

Differences between ECHAM6.3, ICON‐A, ECHAM6.3‐HAM2.3, and ICON‐A‐HAM2.3 (except for parameter settings which will be discussed in Section 2.6 below) are summarized in Table 1. Further details regarding the Max Planck Institute physics package and its implementation in ECHAM6.3 and ICON‐A can be found in Mauritsen et al. (2019) and Giorgetta et al. (2018), respectively.

**Table 1:** Overview of Differences Between Model Versions (Except Parameter Settings)

	ECHAM6.3	ICON‐A	ECHAM6.3‐HAM2.3	ICON‐A‐HAM2.3
dynamical core	spectral transform	grid	spectral transform	grid
turbulent diffusion	TKE scheme	TTE scheme	TKE scheme	TTE scheme
aerosol and cloud				
droplet number	prescribed		computed	
concentration				
cloud microphysics	single moment		double moment	

*Note.* Please refer to Sections 2.1–2.4 for details.

### 2.4 The HAM2.3 Aerosol Module

The Hamburg Aerosol Module (HAM) Version 2.3 (HAM2.3) is described by Tegen et al. (2019). HAM was originally developed by Stier et al. (2005) and was updated to version 2 by Zhang et al. (2012). ECHAM6.3‐HAM2.3 and ICON‐A‐HAM2.3 compute emissions, transport, wet and dry deposition of sulfate, black carbon (BC), organic carbon (OC), sea salt, and mineral dust assuming internally mixed particles within each mode. Nucleation of sulfate aerosol and aerosol growth by deposition of vapors and coagulation of particles are taken into account.

By default, aerosol microphysical processes are treated by the M7 aerosol microphysics module (Vignati et al., 2004). This modal aerosol microphysics parameterization distinguishes between hydrophilic and hydrophobic aerosol. Hydrophilic aerosol is subdivided into four log‐normal modes and hydrophobic aerosol into three modes. Each mode contains one or more species (Table 2). The standard deviation of the modes is fixed. The mass concentration of each species in each mode is carried as a prognostic variable, accounting for 18 prognostic variables. Within each mode, the species are assumed to be internally mixed. Aerosol number for each of the modes accounts for seven prognostic variables. Aerosol water content for the four modes containing hydrophilic aerosol adds another four prognostic variables. For an internal mixture to be considered hydrophilic, it suffices if one of the species is hydrophilic.

**Table 2:** Aerosol Size Modes and Species in the M7 Aerosol Microphysics in HAM (Adopted From Tegen et al., 2019)

Size Mode	Hydrophilic	Hydrophobic
Nucleation $\left(\bar{r}<0.005\;\mu m\right)$	Sulfate	
Aitken $\left(0.005\;\mu m<\bar{r}<0.05\;\mu m\right)$	Sulfate, OC, BC	OC, BC
Accumulation $\left(0.05\;\mu m<\bar{r}<0.5\;\mu m\right)$	Sulfate, OC, BC, sea salt, dust	Dust
Coarse $\left(\bar{r}>0.5\;\mu m\right)$	Sulfate, OC, BC, sea salt, dust	Dust

*Note.* $\bar{r}$ is Number Median Radius.

In M7 aerosols grow due to coagulation, the condensation of sulfuric acid, and aerosol water uptake. Hydrophobic particles can become hydrophilic either by coagulation with hydrophilic particles or due to the condensation of sulfuric acid. Because condensation and coagulation growth increase the mode mean radius and standard deviation, a mode merging algorithm (Vignati et al., 2004) is applied to repartition the particles among the modes and to confine the number median radius of each mode to the range given in Table 2 (Stier et al., 2005). Aerosol water content is computed following Petters and Kreidenweis (2007) as implemented by O’Donnell et al. (2011). Aerosol optical properties are computed based on volume‐weighted averages of the refractive indices and Mie‐scattering size parameters of the individual components including the water content, assuming internal mixing (Stier et al., 2007). For computing aerosol growth due to water uptake, in partially cloud‐covered grid boxes, only clear‐sky relative humidity is taken into account. In fully cloud covered grid boxes, air is assumed to be saturated (Section 2.6 of Stier et al., 2005).

Some aerosol is emitted directly (see Section 2.4.2). This so‐called primary aerosol is emitted in the bulk solid phase, for example, from deserts, or in the liquid phase, for example, from the oceans. Aerosols can also be formed from the gas phase by gas‐to‐particle conversion (secondary aerosol). In HAM2.3, neutral and charged nucleation of sulfuric acid and water (Kazil & Lovejoy, 2007) and nucleation of an organic compound and sulfuric acid via cluster activation (Kulmala et al., 2006; Riipinen et al., 2007) are treated based on the implementation by Kazil et al. (2010). Formation of secondary organic aerosol (SOA) due to biogenic emissions is included as primary OC emissions following AeroCom (Dentener, Kinne, et al., 2006) as in the ECHAM6.3‐HAM2.3 default setup.

Sulfate and gaseous sulfuric acid formation are computed based on the sulfur chemistry of Feichter et al. (1996). Sulfate (${SO}_{4}^{-2}$), and the precursor gases dimethyl sulfide (DMS) and sulfur dioxide (SO_2_) are treated as prognostic variables, taking into account emissions, transport, chemistry, and wet and dry deposition. Oxidant concentrations are prescribed as monthly mean fields based on the Monitoring Atmospheric Composition and Climate (MACC) reanalysis (Inness et al., 2013). Gaseous sulfuric acid can either condense on existing aerosol particles or nucleate to form new particles. Sulfate produced from aqueous phase chemistry is distributed to pre‐existing hydrophilic accumulation mode and coarse mode aerosol particles (Stier et al., 2005; Zhang et al., 2012).

#### 2.4.1 Cloud Microphysics and Radiation Coupling

The two‐moment cloud microphysics scheme (Lohmann & Hoose, 2009; Lohmann & Neubauer, 2018; Lohmann et al., 2007) in ECHAM6.3‐HAM2.3 and ICON‐A‐HAM2.3 computes condensation, evaporation, freezing, melting, deposition, sublimation, autoconversion of cloud droplets to rain drops, accretion of raindrops with cloud droplets and of snow flakes with cloud droplets and ice crystals, aggregation of ice crystals, evaporation of raindrops, melting and sublimation of snowflakes, and the sedimentation of cloud ice (Lohmann & Neubauer, 2018). Droplet activation is parameterized based on Köhler‐theory using the parameterization by Abdul‐Razzak and Ghan (2000) as in Tegen et al. (2019) and Lohmann and Neubauer (2018). The Abdul‐Razzak and Ghan (2000) activation parameterization was originally implemented for use with prognostic HAM aerosol by Stier (2016). A minimum cloud droplet number concentration (CDNC) of 40 cm^−3^ is used in ECHAM6.3‐HAM2.3 and ICON‐A‐HAM2.3; see Hoose et al. (2009) and Neubauer et al. (2019) for discussion. For computing supersaturation, the effect of sub‐grid scale vertical velocity is parameterized as a function of turbulent kinetic energy (TKE) as in Lohmann et al. (1999). The total vertical velocity *w* is computed as the sum of grid‐scale mean vertical velocity $\bar{w}$, and a contribution due to subgrid‐scale vertical velocity as $w=\bar{w}+0.7\sqrt{TKE}$.

Autoconversion of cloud droplets to form rain is computed using the Khairoutdinov and Kogan (2000) parameterization. Heterogeneous freezing takes into account contact and immersion freezing of mineral dust following Lohmann and Hoose (2009) based on Lohmann and Diehl (2006), with modifications by Lohmann and Neubauer (2018). Homogeneous freezing of supercooled solution droplets follows Lohmann and Kärcher (2002). Detrainment from convective cloud is a source of condensate for the stratiform cloud scheme as in Lohmann and Neubauer (2018). Based on the assumption that convective cores cover a much smaller horizontal area than stratiform clouds, only optical properties of stratiform clouds are taken into account in the radiation parameterization.

#### 2.4.2 Emissions

HAM2.3 treats emissions of aerosol (BC, OC, sea salt, and mineral dust) and aerosol precursor gases (SO_2_, DMS) from natural and anthropogenic sources including industry, energy, agricultural waste burning, domestic, air traffic, land traffic, shipping, natural biogenic, forest fires, grass fires, explosive volcanoes, smoldering volcanoes, the land surface (for dust and DMS) and the ocean (for sea salt and DMS). In the default ECHAM6.3‐HAM2.3 and ICON‐A‐HAM2.3 versions, monthly mean anthropogenic and biomass burning emissions are taken from the interpolated Atmospheric Chemistry and Climate Model Intercomparison Project (ACCMIP) and Representative Concentration Pathway (RCP) emission data set which is based on best guess historical emission estimates (Lamarque et al., 2010) until the year 2000. For the years beyond 2000, the data is provided for four different RCP scenarios (van Vuuren et al., 2011). For 2001–2012 we use the RCP8.5 scenario. The original data is available at http://aerocom.met.no/DATA/download/emissions/AEROCOM-II-ACCMIP/ (last access: 17 July 2020). The data is then regridded to the respective horizontal grid using the Climate Data Operators (CDO) software (Schulzweida, 2019). The injection heights for industry (50 m), ships (50 m), and aircraft (fixed model level) are prescribed. Biomass burning injections are vertically distributed following Martin et al. (2010).

Mineral dust emissions are computed taking into account simulated wind speed, soil humidity, and snow cover based on Tegen et al. (2002) with modifications by Cheng et al. (2008) and Heinold et al. (2016). In ECHAM6.3‐HAM2.3 regional correction factors have been applied to the dust emissions (Tegen et al., 2019) in order to match dust emissions estimated by Huneeus et al. (2011). In ICON‐A‐HAM2.3, a globally constant correction factor of 0.9 was set without further tuning. This is close to the constant correction factor (0.86) in ECHAM‐HAM model versions prior to ECHAM6.3‐HAM2.3. Sea salt emissions are parameterized based on Long et al. (2011) taking into account wind speed and also temperature based on Sofiev et al. (2011). DMS emissions are computed taking into account wind speed based on Nightingale et al. (2000). DMS concentrations in sea water are prescribed based on Lana et al. (2011). Volcanic sulfur emissions are taken from Dentener, Stevenson, et al. (2006).

#### 2.4.3 Wet and Dry Deposition

Wet deposition of aerosol by stratiform clouds is parameterized using the scheme by Croft et al. (2010), which takes into account nucleation and size‐dependent impaction in‐cloud scavenging and below‐cloud scavenging based on Croft et al. (2009). For deep convection, scavenging factors are prescribed (Section 2.5). For the trace gases, Henry's law equilibrium is assumed (e.g., Seinfeld & Pandis, 1998). Gravitational sedimentation of aerosol is based on a computation of the Stokes velocity (Seinfeld & Pandis, 1998; Stier et al., 2005). The parameterization of aerosol and trace gas dry deposition (Ganzeveld & Lelieveld, 1995; Ganzeveld et al., 1998) is based on the resistance approach (Wesely, 1989) together with the “big leaf” approximation (e.g., Hicks et al., 1987) for the dry deposition of trace gases on vegetation.

#### 2.4.4 Implementation of HAM2.3 Into ICON‐A

HAM was originally developed as a component for ECHAM. Because ECHAM6.3 and ICON‐A share the Max Planck Institute physics package, adapting HAM2.3 to ICON‐A involved mainly technical tasks. These tasks were facilitated by the modular approach in ECHAM6.3‐HAM2.3‐MOZ1.0 and so‐called Submodel Interface (see below). In recent years, significant effort was made in designing a generic interface between ECHAM, HAM, and the MOZ module for tropospheric and stratospheric chemistry, especially in the framework of the ECHAM6.3‐HAM2.3‐MOZ1.0 development (Schultz et al., 2018). However, in spite of the modular structure, sub‐routines still had to be adapted individually, and all the options in ECHAM6.3‐HAM2.3 are not yet available in ICON‐A‐HAM2.3. ICON‐A‐HAM2.3 currently supports only the default M7 aerosol microphysics.

ICON‐A facilitates the addition of prognostic tracers via a user‐friendly interface. In total, coupling HAM2.3 involves the addition of 34 prognostic variables to the host model, including 29 variables for the M7 aerosol microphysics module, three for the sulfur chemistry, and two additional variables for the two‐moment cloud microphysics. These prognostic variables are advected and are carried through the turbulent mixing parameterization and convective transport. In order to ensure that hydrophilic aerosol and soluble gases are also subjected to scavenging associated with moist convection, the HAM2.3 wet deposition interface is called from the moist convection parameterization. Scavenging in large‐scale stratiform clouds is called from the two‐moment cloud microphysics parameterization via the Submodel Interface. To ensure proper coupling of emissions to boundary layer turbulence, the HAM2.3 emission interface is called from the turbulent mixing parameterization.

**Figure 1:**
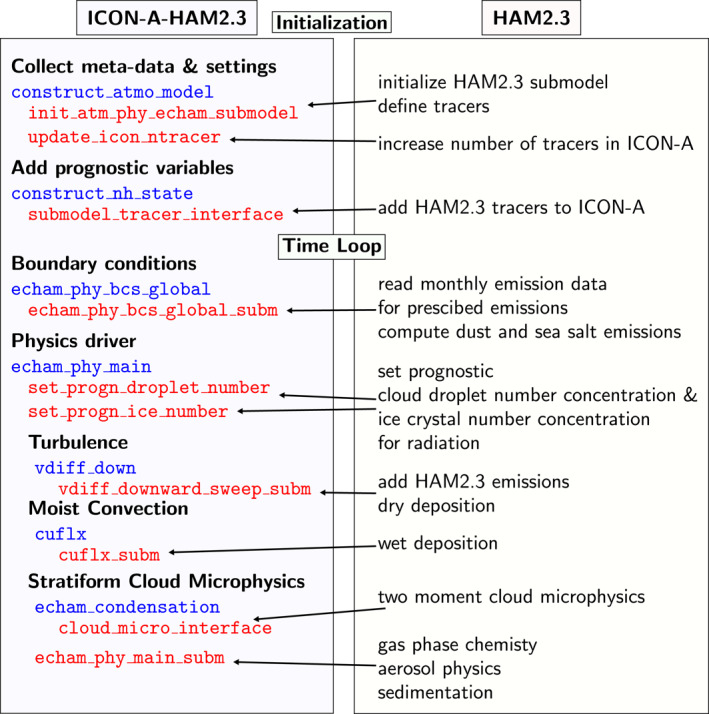
Schematic: Coupling of HAM2.3 to ICON‐A via the Submodel Interface stating the names of the HAM2.3 interface routines (red) and the ICON‐A calling routines/entry points (blue).

The HAM2.3‐specific subroutines that are called from the ICON‐A host model are part of a Fortran 90 module referred to as Submodel Interface. The calling routines, here referred to as entry points, are mainly Fortran 90 subroutines which are included in ICON‐A (icon‐aes‐1.3.00). Figure 1 illustrates several rather technical aspects of the coupling of HAM2.3 to ICON‐A via the Submodel Interface and also summarizes how HAM2.3 is coupled to ICON‐A‐HAM2.3.

### 2.5 Model Setup

The horizontal grid resolution used in the runs in this study is T63 for ECHAM6.3 (e.g., Stevens et al., 2013; Crueger et al., 2018) and R2B4 (around 160 km) for ICON‐A (see Crueger et al., 2018; Giorgetta et al., 2018). The T63 spectral truncation corresponds to a 1.87° × 1.87° grid, with a grid spacing of about 200 × 200 km near the equator (Crueger et al., 2018). ECHAM6.3 uses a vertical hybrid sigma pressure coordinate with 47 levels (Stevens et al., 2013). ICON‐A uses a vertical hybrid sigma height coordinate with 47 levels with a slightly lower resolution in the upper troposphere than ECHAM6.3 (Giorgetta et al., 2018). We simulate 10 years from 2003 to 2012 after a three‐month initial spinup in an Atmospheric Model Intercomparison Project (AMIP) setup with prescribed sea surface temperatures (SSTs) and sea‐ice cover (Taylor et al., 2012) as in the ECHAM6.3‐HAM2.3 reference setup (Neubauer et al., 2019; Tegen et al., 2019). Aerosol emissions are either specified or computed as described in Section 2.4.2. The T63 resolution is still a standard resolution for ECHAM6.3 because it allows users to experiment more freely and because model configurations that are well known and characterized are usually easier to learn from (Mauritsen et al., 2019).

### 2.6 Parameter Settings

Although the ECHAM6.3 and ICON‐A host models both use the Max Planck Institute physics package, the standard parameter settings vary (Table 3). As indicated above, coupling HAM2.3 involves the inclusion of a two‐moment cloud microphysics scheme and subsequent re‐tuning for radiation balance. The corresponding parameter settings in ECHAM6.3‐HAM2.3 and ICON‐A‐HAM2.3 are also presented in Table 3.

**Table 3:** Parameter Settings

	ICON‐A	ICON‐A‐HAM2.3	ECHAM6.3	ECHAM6.3‐HAM2.3
radiation balance tuning (RBT) parameters				
*δp*_min_ (hPa)	0	0	300	300
*α*_r_	15	15	15	10.6
*α*_s_	95	95	95	900
*α*_c_ (10^−4^ s^−1^)	2.5	1.5	1.5	9.0
*ϵ*_*s*_ (10^−3^ m^−1^)	3	3	3	3
*ϵ*_*m*_ (10^−4^ m^−1^)	2	3	1	1
*ϵ*_*p*_ (10^−4^ m^−1^)	2	3	1	2
*ϵ*_*d*_ (10^−4^ m^−1^)	4	4	2	2
*f*_n_	0.2	0.17	0.2	0.2
*γ*_s_	0.968	0.968	0.975	0.975
*γ*_t_	0.8	0.8	0.75	0.75
*β*_l_ (low/mid/high)	0.8/0.4/0.8	0.85/0.45/0.85	0.8/0.4/0.8	0.8/0.4/0.8
*β*_i_	0.8	0.85	0.8	0.7
*τ*_*c*_ (s)	3600	3600	7200	7200
a_ins_	10	10	0	0
Scavenging factors in convective clouds in HAM2.3				
S (modes)		0.10/0.25/0.75/0.85		0.20/0.60/0.99/0.99

*Note.* Minimum pressure difference between cloud top and cloud base before the moist convection parameterization starts to produce rain (*δp*_min_). Scale factors for conversion of cloud water to rain (*α*_r_) and conversion of ice crystals to snow (*α*_s_) in stratiform clouds, rate of conversion of cloud water to rain in convective clouds (*α*_c_), entrainment rate for shallow (*ϵ*_s_), mid‐level (*ϵ*_m_), penetrative (*ϵ*_p_) convective clouds, and downdrafts (*ϵ*_d_). Cloud mass‐flux above level of non‐buoyancy (f_n_) expressed as a fraction of cloud mass‐flux, entrainment rate for shallow convection, critical relative humidity at surface (*γ*_s_) and aloft (*γ*_t_), homogeneity factor for low‐level, mid‐level, and high‐level liquid (*β*_l_) and ice clouds (*β*_i_). Characteristic adjustment time scale for convective clouds (*τ*_c_). Factor in accretion of ice crystals with newly formed snow (a_ins_). Scavenging factor in convective clouds for hydrophilic particles (S) for the nucleation, Aitken, accumulation, and coarse mode.

One change in the parameter settings of the host models pertains to the minimum pressure difference between cloud top and cloud base before the moist convection parameterization starts to produce rain (*δp*_min_). The lack of a depth requirement in ICON‐A implies that penetrative moist convection can precipitate without growing into deep convection. This parameter was ultimately left unchanged from the original ICON‐A value in ICON‐A‐HAM2.3 in order to avoid re‐introducing a long‐standing AOT bias over subtropical oceans (see Section 4.3). Instead, scavenging factors for scavenging associated with the moist convection parameterization have been reduced, partially compensating for the effect of the change of *δp*_min_ between ECHAM6.3 and ICON‐A. Convective clouds can produce rain earlier in ICON‐A‐HAM2.3, and lower scavenging factors counteract the increased scavenging.

On the one hand, based on Köhler theory, one expects internally mixed coarse and accumulation mode particles to be activated when entering the cloud through the cloud base. On the other hand, this is not necessarily the case for lateral entrainment. Based on a case study investigating deep convective clouds, whose cores tend to be comparatively undiluted by lateral entrainment compared to shallow convective clouds, Yang et al. (2015) derived scavenging efficiencies for submicron aerosol total dry mass of 0.81 and 0.83, combining observations with two different modeling approaches. Furthermore, Croft et al. (2012) found the standard scavenging factors which are applied in ECHAM6‐HAM2.3 resulted in more efficient aerosol scavenging in convective clouds compared to a more sophisticated treatment of aerosol scavenging. Here, we reduced the scavenging factors from 0.99 for coarse and accumulation mode aerosol to 0.85 and 0.75, respectively (Table 3). Reduced scavenging factors in convective clouds for nucleation and Aitken mode aerosol in Table 3 are consistent with observations by Henning et al. (2004).

### 2.7 Setup of Tuning Sensitivity Runs

The effect of reducing the scavenging factors for internally mixed aerosol associated with convective clouds in ICON‐A‐HAM2.3 on AOT can be assessed by comparing the AOT from a standard ICON‐A‐HAM2.3 run to the AOT from a sensitivity run in which the scavenging factors (S in Table 3) were set to the original values from ECHAM6.3‐HAM2 (also stated in Table 3). Because the radiation balance tuning parameters in this sensitivity run are set to the standard values used in ICON‐A‐HAM2.3, this sensitivity run is referred to as ICON‐A‐HAM2.3 RBT run, where RBT stands for radiation balance tuned (compare Table 4). The setup of the ICON‐A‐HAM2.3 RBT run is identical to that of ICON‐A‐HAM2.3, except for the reduction of scavenging factors.

**Table 4:** ICON‐A‐HAM2.3 Sensitivity Runs With Original Scavenging Factors as in ECHAM6.3‐HAM2.3 (see Table 3)

Label	Parameter settings	Description
RBT	ICON‐A‐HAM2.3	radiation balance tuning parameters set as in
		ICON‐A‐HAM2.3, but still using the original
		scavenging factors from ECHAM6.3‐HAM2.3
NT	ICON‐A	not tuned: original radiation balance tuning
		parameters from ICON‐A and original
		ECHAM6.3‐HAM2.3 scavenging factors
CPC	NT, but with ICON‐A‐HAM2.3 *α*_c_	sensitivity runs to investigate the effect of
ENM	NT, but with ICON‐A‐HAM2.3 *ϵ*_*m*_	ICON‐A‐HAM2.3 radiation balance tuning
ENP	NT, but with ICON‐A‐HAM2.3 *ϵ*_*p*_	parameters on AOT
MFT	NT, but with ICON‐A‐HAM2.3 *f*_n_	(varying one parameter at a time)
DLV	NT, but with ECHAM6.3‐HAM2.3 *δp*_min_	sensitivity runs to investigate the effect of
TDLV	NT, but with *δp*_min_ set to 100 hPa	different radiation balance tuning parameters
RAC	NT, but with ECHAM6.3‐HAM2.3 *α*_*r*_	in host models on AOT
SAC	NT, but with ECHAM6.3‐HAM2.3 *α*_*s*_	
CPC2	NT, but with ECHAM6.3‐HAM2.3 *α*_c_	
ENM2	NT, but with ECHAM6.3‐HAM2.3 *ϵ*_*m*_	
END	NT, but with ECHAM6.3‐HAM2.3 *ϵ*_*d*_	
RHC	NT, but with ECHAM6.3‐HAM2.3 *γ*_*s*_, *γ*_*t*_	
TSC	NT, but with ECHAM6.3‐HAM2.3 *τ*_*c*_	
INS	NT, but with ECHAM6.3‐HAM2.3 a_ins_	
CPC3	ICON‐A‐HAM2.3 but with	sensitivity runs to investigate the effect of
	ECHAM6.3‐HAM2.3 *α*_c_	*α*_c_ on ERFari + aci

*Note.* Parameter values are given in Table 3.

The ICON‐A‐HAM2.3 NT run, where NT stands for “not tuned,” in Table 4 is a run in which the original radiation balance tuning parameters from ICON‐A and original ECHAM6.3‐HAM2.3 scavenging factors were used in ICON‐A‐HAM2.3. In other words, the ICON‐A‐HAM2.3 NT setup is a setup in which HAM2.3 has been coupled to ICON‐A without either radiation balance tuning or an adjustment of the scavenging factors. By comparing ICON‐A‐HAM2.3 RBT to ICON‐A‐HAM2.3 NT one can assess the effect of radiation balance tuning in ICON‐A‐HAM2.3. As indicated above, this tuning was necessary after coupling HAM2.3 to ICON‐A in order to reduce the radiative imbalance at the top of the atmosphere in ICON‐A‐HAM2.3.

Apart from the RBT and NT sensitivity runs, Table 4 contains two additional sets of sensitivity runs. These sets are separated by horizontal lines in Table 4. The first set (CPC to MFT) was designed to understand the effect of changing individual radiation tuning parameters from their original values in ICON‐A to the values in ICON‐A‐HAM2.3. The second set of sensitivity runs in Table 4 addresses differences between ICON‐A‐HAM2.3 and ECHAM6.3‐HAM2.3. It was motivated by a desire to understand the unexpected absence of a long‐standing AOT bias over tropical oceans after coupling HAM2.3 to ICON‐A. This bias had been present in the earlier versions of ECHAM‐HAM, which had applied different radiation balance tunings compared to ECHAM6.3‐HAM2.3. Therefore, in this second set of runs, we focus on comparing the effect of radiation tuning parameters in the host models. In order to understand whether the absence of the AOT bias could be caused by a tuning parameter setting that was changed in the host model, and in order to decide which parameter setting or combination of parameter settings may be responsible, in the second set of runs, we performed sensitivity runs for a total of eight different radiation tuning parameters.

### 2.8 Setup of Runs to Compute Effective Radiative Forcing

In order to compute the effective radiative forcing due to aerosol‐radiation and aerosol‐cloud interactions (ERFari + aci) we performed 10‐year runs (plus 3 months initial spinup) with 1850 (pre‐industrial) anthropogenic aerosol emissions with ICON‐A‐HAM2.3 and with ECHAM6.3‐HAM2.3. ERFari + aci was then computed as the difference of top‐of‐atmosphere (TOA) net radiation between the near present‐day (2003–2012) run and this pre‐industrial run. In order to investigate the reason for a slightly higher ERFari + aci in ICON‐A‐HAM2.3 compared to ECHAM6.3‐HAM2.3, we performed three additional pre‐industrial runs with ICON‐A‐HAM2.3. The first of these additional pre‐industrial runs was performed using the NT setup from Table 4. The second additional pre‐industrial run was performed with *δp*_min_ = 300 hPa as in the DLV setup because this parameter was found to largely explain the difference of simulated AOTs over the subtropical oceans between ICON‐A‐HAM2.3 and ECHAM6.3‐HAM2.3. In an additional sensitivity run, we increased the rate of conversion of cloud water to rain in convective clouds (*α*_c_) from its default ICON‐A‐HAM2.3 value of 1.5 10^−4^ s^−1^ to 9.0 10^−4^ s^−1^, which corresponds to the default value of *α*_c_ in ECHAM6.3‐HAM2.3. Our original hypothesis was that ERFaci may perhaps be sensitive to *α*_c_ because the setting of *α*_c_ can affect the partitioning between stratiform and convective precipitation. Because computing ERFari + aci requires a set of two comparable runs consisting of a near present‐day and a pre‐industrial run, we performed an additional near present‐day (2003–2012) run for this setup as well. These additional sensitivity runs again used the scavenging factors from ECHAM6.3‐HAM2.3.

## 3 Observational and Re‐Analysis Data

Satellite retrievals of AOT are taken from the MODerate Resolution Imaging Spectroradiometer MODIS collection 6.1 level‐3 monthly data for the Aqua satellite (MYD08_M3) (Levy et al., 2013; Platnick, King, et al., 2015). Unless indicated otherwise, all gridded data, including data from ICON‐A‐HAM2.3, were interpolated to the ECHAM6.3 T63 resolution in order to facilitate computation and visualization of the differences between simulation results and gridded observational data. Only grid points for which MODIS data is available were used for the comparison. Daily ground‐based sun‐photometer observations of AOT at 590 sites from the AErosol RObotic NETwork (AERONET; https://aeronet.gsfc.nasa.gov, last access: 29 November 2019, Holben et al., 1998), for which at least one month of data with observations on at least half of the days of this month was available, were averaged to monthly data. Because nudging (e.g., Tegen et al., 2019) has not yet been implemented, no hourly/daily collocation in time was performed for the AERONET data. In‐situ observations of sea salt by the Particle Analysis by Laser Mass Spectrometry (PALMS; Murphy et al., 2006) and black carbon by the single‐particle soot photometer (SP2; Schwarz et al., 2006) over the tropical Pacific Ocean are averaged from the merged Atmospheric Chemistry, Trace Gases, and Aerosols (ATom; Wofsy et al., 2018) data. Top of the atmosphere radiation is taken from the Clouds and the Earth's Radiant Energy System (CERES) Energy Balanced and Filled (EBAF) Top‐of‐Atmosphere (TOA) Edition‐4.1 (Loeb et al., 2018) data set. For precipitation, the Global Precipitation Climatology GPCP V2.3 (Adler et al., 2003, 2018) is used.

Climatological biases of meteorological variables for computing the combined multivariable relative biases discussed in Section 4.9 are based on various datasets as described by Crueger et al. (2018): Air pressure at sea level, wind stresses, temperature at 850 hPa, stationary waves at 500 hPa (geopotential), zonal mean temperature and zonal mean zonal wind is taken from European Centre for Medium‐Range Weather Forecasts (ECMWF) interim re‐analysis (ERA‐Interim; Dee et al., 2011). Outgoing TOA radiation is again from CERES‐EBAF, column water vapor content from the National Aeronautics and Space Administration Water Vapor Project (NVAP; Randel et al., 1996), precipitation over ocean from the Hamburg Ocean Atmosphere Parameters and Fluxes from Satellite (HOAPS; Andersson et al., 2010), precipitation over land from GPCP V2.2 as in Crueger et al. (2018), and surface land temperature from the Climatic Research Unit (CRU), University of East Anglia, land station temperature data set (CRUTEM4; Jones et al., 2012).

## 4 Results

### 4.1 Evaluation of Aerosol Optical Thickness (AOT) With Satellite Observations and Initial Discussion of Differences Between ECHAM6.3‐HAM2.3 and ICON‐A‐HAM2.3

**Figure 2:**
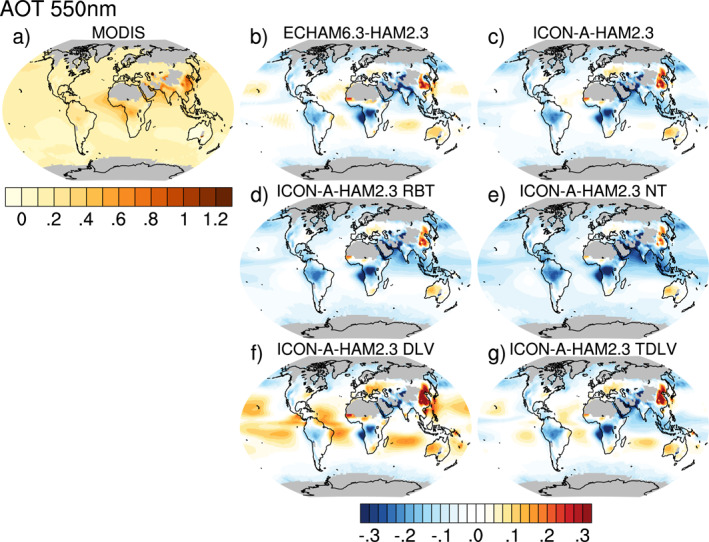
Aerosol optical thickness (AOT) at 550 nm retrieved from MODIS measurements on board the Aqua satellite for the years 2003–2012 (a). Difference between model simulated AOT and and MODIS AOT (b)–(g), for ECHAM6.3‐HAM2 (b), for ICON‐A‐HAM2.3 (c), for ICON‐A‐HAM2.3 RBT (sensitivity run tuned for radiation balance, but with scavenging factors from ECHAM6.3‐HAM2.3) (d), for ICON‐A‐HAM2.3 NT (not tuned for radiation balance, i.e., original ICON‐A radiation balance tuning parameters, and also scavenging factors from ECHAM6.3‐HAM2.3) (e), for ICON‐A‐HAM2.3 DLV (as ICON‐A‐HAM2.3 NT, but with minimum pressure difference between cloud top and cloud base before the moist convection parameterization starts to produce rain (*δp*_min_ = 300 hPa) as in ECHAM6.3) (f), and for ICON‐A‐HAM2.3 TDLV (as ICON‐A‐HAM2.3 NT, but with *δp*_min_ set to 100 hPa) (g).

Aerosol optical thickness (AOT) at 550 nm from MODIS (Figure 2a) is compared to AOT from ECHAM6.3‐HAM2.3 and ICON‐A‐HAM2.3 in Figures 2b and 2c. Both models show negative (or low) biases over the tropical rain forests and over the northern Indian Ocean, the Red Sea, and the Persian Gulf. These negative biases are more negative in ICON‐A‐HAM2.3 compared to ECHAM6.3‐HAM2.3. However, unlike ICON‐A‐HAM, ECHAM6.3‐HAM2.3 shows a positive (or high) bias over the subtropical oceans and over parts of the tropical oceans in the main subsidence regions outside the Intertropical Convergence Zone and South‐Pacific Convergence Zone, especially in the descending branch of the Walker Circulation off the coast of Peru in the southern tropical East Pacific.

**Figure 3:**
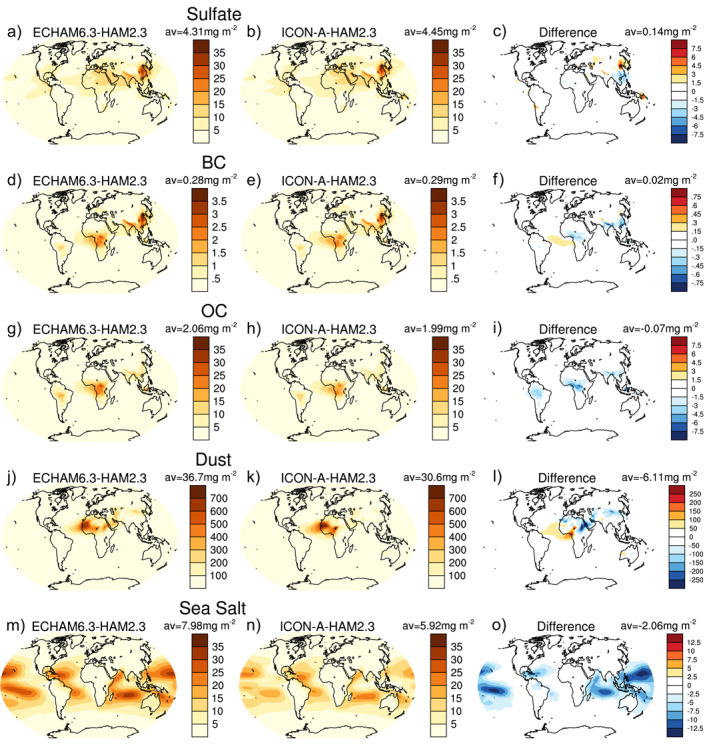
Column‐integrated aerosol burden of sulfate (a‐c), black carbon (d‐f), organic carbon (d‐f), dust (j‐l), and sea salt (m‐o) aerosol for the years 2003–2012 from ECHAM6.3‐HAM2.3 (left column) and ICON‐A‐HAM2.3 (middle) and the difference between ICON‐A‐HAM2.3 and ECHAM6.3‐HAM2.3 (right column).

In order to investigate which aerosol‐related process is mainly responsible for the lower AOTs over the subtropical oceans in ICON‐A‐HAM2.3 compared to ECHAM6.3‐HAM2.3, we first compare the differences in column‐integrated aerosol burdens in Figure 3 and then discuss contributions of individual aerosol processes. Of all the differences in aerosol burdens investigated in Figure 3, only the sea salt aerosol burden difference in Figure 3o shows a pattern that is fairly compatible with the pattern of lower AOT over the subtropical oceans. Overall, the global mean column‐integrated sea salt aerosol burden is about 26% smaller in ICON‐A‐HAM2.3 compared to ECHAM6.3‐HAM2.3 (Figures 3m and 3n).

**Figure 4:**
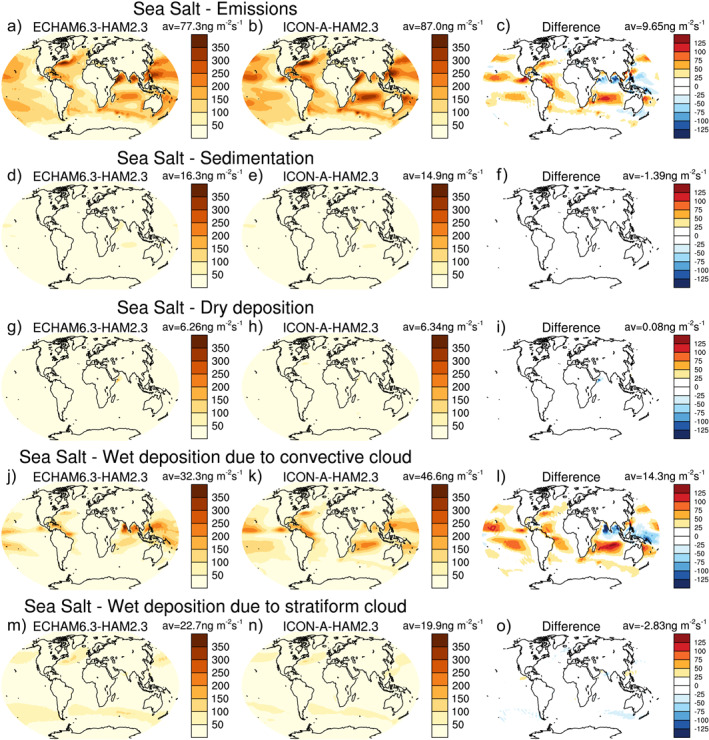
Sea salt aerosol emission (a‐c), sedimentation (d‐f), dry deposition (d‐f), wet deposition due to convective cloud (j‐l), and wet deposition due to stratiform cloud (m‐o) fluxes for the years 2003–2012 from ECHAM6.3‐HAM2.3 (left column) and ICON‐A‐HAM2.3 (middle) and the difference between ICON‐A‐HAM2.3 and ECHAM6.3‐HAM2.3 (right column).

The smaller column‐integrated sea salt aerosol burden in ICON‐A‐HAM2.3 compared to ECHAM6.3‐HAM2.3 over the subtropical oceans cannot be explained by a difference in sea salt emissions (Figures 4a–4c), because the differences in emissions point in the opposite direction. In the absence of other factors, higher sea salt emissions in ICON‐A‐HAM2.3 (Figure 4b) compared to ECHAM6.3‐HAM2.3 (Figure 4a) would act to increase of AOTs over subtropical oceans in ICON‐A‐HAM2.3 relative to ECHAM6.3‐HAM2.3. The higher sea salt emissions in ICON‐A‐HAM2.3 relative to ECHAM6.3‐HAM2.3 over the subtropical oceans are consistent with higher near‐surface wind speed in ICON‐A‐HAM2.3 compared to ECHAM6.3‐HAM2.3 in these regions (see Figure S1a–S1c in Supporting Information S1). ICON‐A also shows higher wind speeds over subtropical oceans compared to ECHAM6.3 (Figures S1d–S1f in Supporting Information S1), but the difference between ICON‐A and ECHAM6.3 is smaller than the difference between ICON‐A‐HAM2.3 and ECHAM6.3‐HAM2.3, which is likely related to different radiation balance tuning parameters.

The higher sea salt aerosol emissions over the subtropical oceans in ICON‐A‐HAM2.3 compared to ECHAM6.3‐HAM2.3 are, however, more than compensated by more efficient wet deposition due to convective clouds in ICON‐A‐HAM2.3 (Figure 4l). The reduction of the low AOT bias over the subtropical oceans in ICON‐A‐HAM2.3 can thus be attributed to more efficient wet deposition due to convective clouds and not to a reduction in emissions. In Section 4.3 below, we will argue that the reduction of the positive AOT bias over subtropical oceans can indeed be linked to the change of a particular parameter setting in the parameterization of convective clouds.

Figure 4l shows that although wet deposition due to convective cloud is more active over the subtropical oceans in ICON‐A‐HAM2.3 compared to ECHAM6.3‐HAM2.3, several regions where convective precipitation is particularly strong, such as the South Pacific Warm pool and the Indian Ocean close to India show the opposite, that is, less active wet deposition due to convective cloud in ICON‐A‐HAM2.3. This issue will be further discussed in Section 4.3, where we investigate the sensitivity of the simulated AOT to parameter settings in the host model.

The positive AOT bias over Eastern China extends further northward in ICON‐A‐HAM2.3 compared to ECHAM6.3‐HAM2.3 (Figures 2b and 2c). This is consistent with a higher sulfate burden in Northeast China in ICON‐A‐HAM2.3 compared to ECHAM6.3‐HAM2.3 (Figure 3c). A reason for this lies in an increase in liquid phase sulfate production in ICON‐A‐HAM2.3 compared to ECHAM6.3‐HAM2.3 (Figures S2a–S2c in Supporting Information S1). This increase in liquid phase sulfate production is most likely caused by more stratiform cloud liquid water being available in ICON‐A‐HAM2.3 compared to ECHAM6.3‐HAM2.3 in this region (Figures S3a–S3c in Supporting Information S1). A larger sink term for sulfate aerosol in this region due to wet deposition by stratiform cloud in ICON‐A‐HAM2.3 compared to ECHAM6.3‐HAM2.3 (Figure S4o in Supporting Information S1) is also consistent with more efficient sulfate production in the liquid phase.

In and around northern India, a much less negative AOT bias is found in ICON‐A‐HAM2.3 compared to ECHAM6.3‐HAM2.3. Here again, the sulfate burden is higher in ICON‐A‐HAM2.3 than in ECHAM6.3‐HAM2.3 (Figure 3c). Again, this is likely related to more liquid phase sulfate production (Figure S2c in Supporting Information S1) and a higher stratiform cloud liquid water path (Figure S3c in Supporting Information S1). The sulfate emissions in Figure S4 in Supporting Information S1 are based on identical input data in ICON‐A‐HAM2.3 and ECHAM6.3‐HAM2.3. Regridding the emissions from the ICON‐A‐HAM2.3 horizontal grid to the ECHAM6.3‐HAM2.3 T63 horizontal grid in Figure S4b in Supporting Information S1 (using a conservative regridding algorithm for the sake of computing difference maps) results in some noise of limited amplitude in the difference plot (Figure S4c in Supporting Information S1). A distinct pattern that likely explains the difference in AOTs over either North Eastern China or north India is absent in Figure S4c.

A stronger negative AOT bias over the African and South American tropical rain forests in ICON‐A‐HAM2.3 compared to ECHAM6.3‐HAM2.3 can be attributed to more efficient wet deposition due to convective clouds of OC and BC aerosol (Figures S5l and S6l in Supporting Information S1), with especially large differences in the Congo. As above, this points to a parameter setting in the deep convection parameterization being responsible for a larger bias in ICON‐A‐HAM2.3. The overall negative AOT bias in both ICON‐A‐HAM2.3 and ECHAM6.3‐HAM2.3 over the tropical rain forests is very likely at least in part caused by uncertainties in the biomass burning emissions (compare Figure 3f of Tegen et al., 2019).

**Figure 5:**
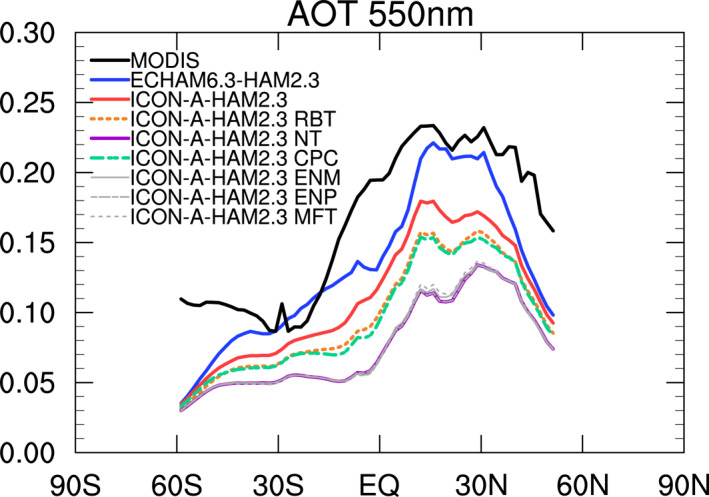
Zonal mean AOT from MODIS, ECHAM6.3‐HAM2.3, ICON‐A‐HAM2.3, and various ICON‐A‐HAM2.3 sensitivity runs (see Table 4).

In the zonal mean AOT in ECHAM6.3‐HAM2.3 (Figure 5), at low latitudes, the high AOT biases over oceanic subsidence regions partially compensate low AOT biases over land regions, especially the South American and the African tropical rain forests (Figure 2b). In ICON‐A‐HAM2.3, on the other hand, zonal mean AOT is clearly underestimated, mainly due to the absence of the high bias over the subtropical oceans.

**Figure 6:**
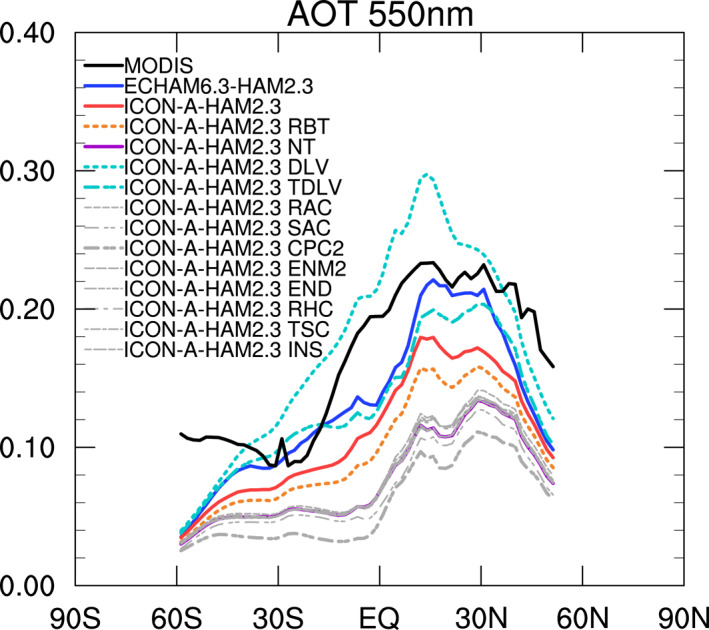
As Figure 5 but for a different set of ICON‐A‐HAM2.3 sensitivity runs (see Table 4).

A negative AOT bias over the west Pacific warm pool is larger in ICON‐A‐HAM2.3 (Figure 2c) than in ECHAM6.3‐HAM2.3 (Figure 2b). However, in the zonal mean for ICON‐A‐HAM2.3 in Figure 5, the negative bias over the west Pacific warm pool is partially compensated by a small positive bias over the Atlantic Ocean in the Saharan outflow (Figure 2c). The largest differences in the zonal mean AOT between ICON‐A‐HAM2.3 and ECHAM6.3‐HAM2.3 (Figure 5) are, however, found in the subtropics where positive and negative biases partially compensate each other in the zonal mean in ECHAM6.3‐HAM2.3. Simulated AOT over eastern China and Australia is higher than the MODIS AOT in both models (Figures 2b and 2c).

The global mean AOT at 550 nm for the years 2003–2012 is 0.13 in ECHAM6.3‐HAM2.3 and 0.11 in ICON‐A‐HAM2.3 (computed based on monthly averaged AOTs at grid points where monthly averaged MODIS AOTs are available). It is 0.16 based on MODIS collection 6.1 level‐3 data. For comparison, Kinne et al. (2006) reported simulated global mean AOTs of 0.11–0.14 for AeroCom models (with 20 different aerosol modules) while a satellite composite yielded a global mean AOT of about 0.15.

### 4.2 Sensitivity of Aerosol Optical Thickness (AOT) to Radiation Balance Tuning

In order to understand the underlying reason for the differences between the AOT from ICON‐A‐HAM2.3 and ECHAM6.3‐HAM2.3 over the subtropical ocean, and also in order to assess the influence of radiation balance tuning on simulated AOT, we performed several sensitivity runs as described in Section 2.5. We will start out by addressing the influence of radiation balance tuning of ICON‐A‐HAM2.3 on simulated AOT. For this purpose, we compare the AOT bias from the ICON‐A‐HAM2.3 RBT run in Figure 2d to the AOT bias from the ICON‐A‐HAM2.3 NT run in Figure 2e. Please recall that the NT run uses the original radiation balance tuning parameters from ICON‐A and the original scavenging factors from ECHAM6.3‐HAM2.3. The RBT run uses the final radiation balance tuning parameters from ICON‐A‐HAM2.3 and the original scavenging factors from ECHAM6.3‐HAM2.3. As indicated above, the radiation balance tuning was performed after coupling HAM2.3 to ICON‐A in order to reduce the radiative imbalance at the top of the atmosphere in ICON‐A‐HAM2.3. Although radiation balance tuning was performed disregarding effects on AOT, on the whole it brings the ICON‐A‐HAM2.3 AOT closer to the MODIS AOT, as evidenced by the smaller biases in Figure 2d compared to Figure 2e. The difference between the bias in the ICON‐A‐HAM2.3 run in Figure 2c and the bias in the ICON‐A‐HAM2.3 RBT sensitivity run in 2d illustrates the effect of decreasing the scavenging factors for convective clouds in ICON‐A‐HAM2.3.

The reason for the higher AOT in the radiation balance tuned ICON‐A‐HAM2.3 RBT run compared to the untuned ICON‐A‐HAM2.3 NT run (Table 4) is explored in Figure 5. The largest AOT increase is due to decreasing the autoconversion rate of cloud droplets to form rain in the convective parameterization, *α*_c_, in ICON‐A‐HAM2.3 RBT. This can be seen by comparing the ICON‐A‐HAM2.3 NT and the ICON‐A‐HAM2.3 CPC sensitivity run. The setup of the CPC run is identical to the NT run, except that *α*_c_ has been decreased from 2.5 × 10^−4^ s^−1^ to 1.5 × 10^−4^ s^−1^. A decrease of *α*_c_ results in slower warm rain formation. A reason for reducing *α*_c_ has been that satellite observations suggest a lower warm rain frequency compared to model simulations (Mülmenstädt et al., 2015, 2020; Suzuki et al., 2015). In addition to reducing *α*_c_, increasing the entrainment rates for mid‐level (*ϵ*_m_) and penetrative (*ϵ*_p_) convective clouds contributes to a higher AOT, as evidenced by the differences between the ENM, ENP, and the NT run, but this effect is smaller compared to the effect of changing *α*_c_.

### 4.3 Sensitivity of Aerosol Optical Thickness (AOT) to Parameter Settings in the Host Model

The reason for the absence of the high AOT bias over the subtropical ocean in ICON‐A‐HAM2.3 can be understood by comparing the AOT bias from the ICON‐A‐HAM2.3 DLV run in Figure 2f and the AOT bias from the ICON‐A‐HAM2.3 TDLV run in Figure 2g to the AOT bias from the ECHAM6.3‐HAM2.3 run in Figure 2b. In the DLV run, the minimum pressure difference between cloud top and cloud base before the moist convection parameterization starts to produce rain (*δp*_min_) was increased from 0 hPa to 300 hPa. The 0 hPa is the default value of *δp*_min_ in ICON‐A. This value was left unchanged in ICON‐A‐HAM2.3. The 300 hPa is the default value of *δp*_min_ in ECHAM6.3. This value was left unchanged in ECHAM6.3‐HAM2.3. Increasing *δp*_min_ to 100 hPa instead of 300 hPa (Figure 2g) yields a similar AOT bias to the one in ECHAM6.3‐HAM2.3.

**Figure 7:**
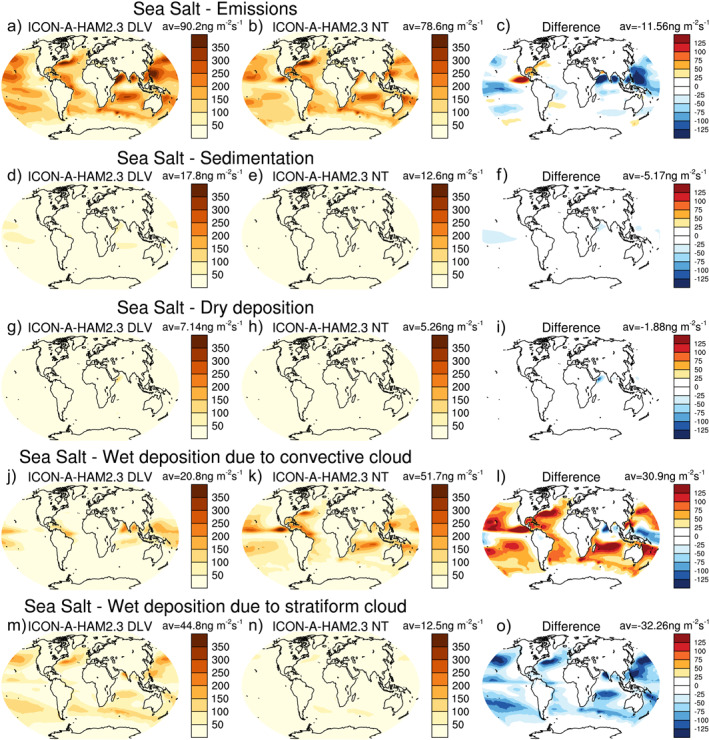
As Figure 4 but for the ICON‐A‐HAM2.3 DLV sensitivity run (left column) and the ICON‐A‐HAM2.3 3 NT sensitivity run (middle) and the difference between ICON‐A‐HAM2.3 NT and ICON‐A‐HAM2.3 DLV (right column).

**Figure 8:**
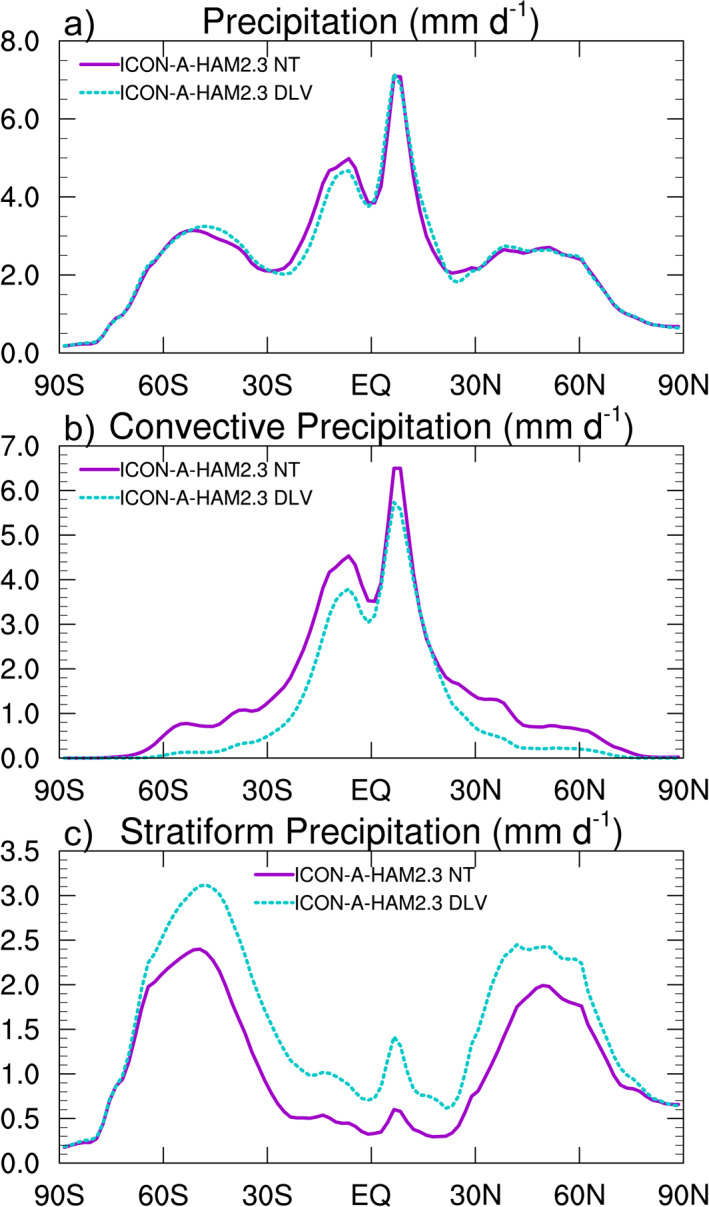
Partitioning of total precipitation (a) into convective (b) and large‐scale stratiform (c) precipitation in the ICON‐A‐HAM2.3 NT run and a comparable sensitivity run with *δp*_min_ = 300 hPa.

The effect of setting *δp*_min_ to 0 hPa in ICON‐A‐HAM2.3 NT instead of setting it to 300 hP in ICON‐A‐HAM2.3 DLV on sea salt is investigated in Figure 7. Please note, that in Figure 7 the ICON‐A‐HAM2.3 DLV run (left column), which uses the *δp*_min_ setting from ECHAM6.3‐HAM2.3, is used as the reference. The ICON‐A‐HAM2.3 NT run is shown in middle column (The corresponding figures for other aerosol species are shown in Figures S8–S11 in Supporting Information S1.) Everything else being equal, setting *δp*_min_ to 0 hPa as in ICON‐A‐HAM2.3 decreases sea salt emissions, increases wet deposition due to convective cloud over the subtropical oceans, and decreases wet deposition due to stratiform cloud, especially in the mid‐altitude storm tracks. The shift from wet deposition due to stratiform cloud to wet deposition due to convective cloud is consistent with a shift from stratiform to convective precipitation when *δp*_min_ is reduced (Figure 8).

Setting *δp*_min_ to 0 hPa increases wet deposition due to convective cloud over the subtropical oceans, but decreases wet deposition in the south west Pacific Warm Pool and around India over the Indian Ocean (Figure 7l), where deep convection contributes a larger fraction to the overall rainfall. Over the subtropical ocean, net large‐scale descent tends to inhibit deep convection. Frequent deep convection over the Pacific Warm Pool and in the northern Indian Ocean is associated with lower tropospheric convergence and net large‐scale average ascent in these regions.

For regions with frequent deep convection, increasing or reducing the *δp*_min_ threshold has a different effect than it does for regions where deep convection is less common. Figure S12 in Supporting Information S1 suggests that removing the *δp*_min_ threshold results in convective precipitation becoming less concentrated in the ascent regions. This is consistent with increased water vapor removal by convection that starts to produce rain already earlier outside these main ascent regions (as evidenced by increased convective precipitation in areas outside the main ascent region in Figure S12c in Supporting Information S1). This also helps to explain decreased wet deposition due to convective cloud in regions of strong net ascent and deep convection (Figure 7l) and can ultimately explain why wet deposition due to convective cloud reacts differently to changing *δp*_min_ over the subtropics than it does in regions of strong lower tropospheric convergence and net ascent. For *δp*_min_ = 0 the increase in convective wet deposition is not fully compensated by the decrease in stratiform wet deposition in subtropical ocean regions.

The decrease in wet deposition due to stratiform clouds that results from setting *δp*_min_ to 0 hPa everything else being equal in Figure 7o is largely compensated in the final ICON‐A‐HAM2.3 (Figure 4o), while the difference in wet deposition due to convective cloud is not (compare Figure 7o to Figure 4o). The smaller difference in emissions is also compensated. In addition to differences between the host models, radiation balance tuning choices and changed scavenging factors play a role for AOTs as well, as discussed above in relation to Figure 5.

The similarity between the AOT bias patterns in the ICON‐A‐HAM2.3 DLV and TDLV sensitivity runs (Figures 2f and 2g) to the AOT bias pattern in ECHAM6.3‐HAM2.3 (Figure 2b) arises as a consequence of adjusting *δp*_min_ toward the value used in ECHAM6‐HAM2.3. Together with the findings that AOT differences over the subtropical ocean can indeed be linked to wet deposition of sea salt associated with convective cloud and that wet deposition of sea salt associated with convective cloud is sensitive to *δp*_min_, this suggests that the absence of the AOT bias over the subtropical ocean in ICON‐A‐HAM2.3 is explained by different parameter settings in the host models.

Likewise, a comparison between the bias from the ICON‐A‐HAM2.3 NT run in Figure 2e and the bias from the corresponding ICON‐A‐HAM2.3 DLV sensitivity run with *δp*_min_ = 300 hPa in Figure 2f suggests that more negative AOT biases with respect to MODIS AOTs over the tropical rain forests and over the northern Indian Ocean, the Red Sea, and the Persian Gulf in ICON‐A‐HAM2.3 than in ECHAM6.3‐HAM2.3 can also be attributed to the change of *δp*_min_. However, low biases are found even in the DLV run. Setting *δp*_min_ to 300 hPa in the DLV run introduces large positive AOT biases over the subtropical oceans, while it has a comparatively smaller effect on reducing the low biases over the tropical rain forests and elsewhere.

Other differences in the parameter settings between ICON‐A‐HAM2.3 and ECHAM6.3‐HAM2.3 have a comparably minor effect on the zonal mean AOT (Figure 6). A notable exception is the setting of the rate of conversion of cloud water to rain in convective clouds, *α*_*c*_, which was increased from 2.5 10^−4^s^−1^ in the ICON‐A‐HAM2.3 NT run to 9.0 10^−4^s^−1^ in the ICON‐A‐HAM2.3 CPC2 run. Increasing *α*_*c*_ lowers the zonal mean AOT as evidenced by the difference between the zonal mean AOT in the ICON‐A‐HAM2.3 NT and the ICON‐A‐HAM2.3 CPC2 run Figure 6 and also as evidenced by the difference between the ICON‐A‐HAM2.3 CPC and the ICON‐A‐HAM2.3 NT run in Figure 5, where it was lowered from 2.5 10^−4^s^−1^ in the ICON‐A‐HAM2.3 NT run to 1.5 10^−4^s^−1^ in the ICON‐A‐HAM2.3 CPC run. The results in Figure 6 and Figure 5 indicate that an AOT increase that results from lowering *α*_*c*_ from 9.0 10^−4^s^−1^ in ECHAM6.3‐HAM2.3 to 1.5 10^−4^s^−1^ in the final ICON‐A‐HAM2.3 partially counteracts the zonal mean AOT increase that results from setting *δp*_min_ to 0 hPa.

In order to prevent re‐introducing the high AOT bias with respect to MODIS AOTs over subtropical oceans into ICON‐A‐HAM2.3, we left *δp*_min_ unchanged from the ICON‐A default value. Consequently, the low bias of the zonal mean and the global mean AOT is stronger in ICON‐A‐HAM2.3 than in ECHAM6.3‐HAM2.3. With respect to the zonal mean and the global mean AOT, reducing the high AOT bias over subtropical oceans unmasks the low biases elsewhere which are readily apparent in Figure 2c.

The smaller AOT bias in and around northern India in ICON‐A‐HAM2.3 compared to ECHAM6.3‐HAM2.3 and also that the positive AOT bias over Eastern China extends further northward in ICON‐A‐HAM2.3 compared to ECHAM6.3‐HAM2.3 can neither be attributed to setting *δp*_min_ to 0 nor to radiation balance tuning based on Figure 2, nor do several other differences in radiation balance tuning parameters between ICON‐A‐HAM2.3 and ECHAM6.3‐HAM2.3 readily explain these differences (Figure S13 in Supporting Information S1). Additional detailed analysis of this particular region would be required to fully understand the reason for these particular differences, which is beyond the scope of this study.

### 4.4 Comparison of AOT With AERONET Ground‐Based Observations

**Figure 9:**
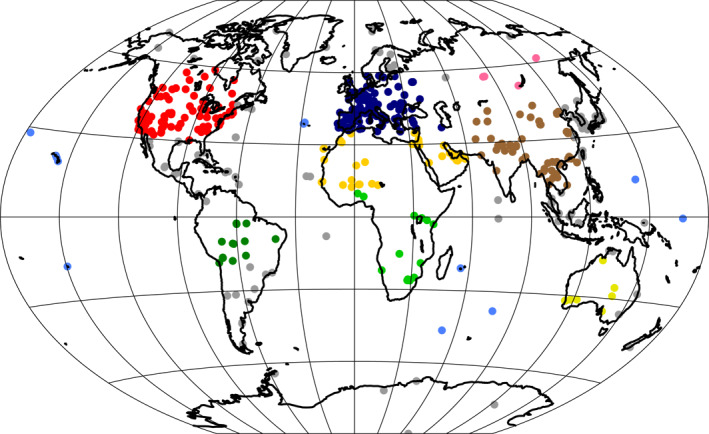
Locations of AERONET stations grouped into regions North America (red), South America (dark green), Europe and Western Mediterranean Sea coastal (dark blue), North Africa and Arabian Peninsula (yellow), South Africa (green), East Asia (brown), Siberia (pink), Australia (lemon), oceanic regions (blue), and mixed regions (gray).

In addition to comparing AOT to MODIS satellite retrievals, we compare monthly mean AOT at AERONET ground stations for which at least 1 month of data with observations on at least half of the days of this month were made. A map of these stations is shown in Figure 9. The map includes all the stations for which the condition is fulfilled. Because the Mauna Loa station is located on a volcano that is too small to be fully resolved at the present grid resolutions, we omitted this station from the analysis. Because the actual weather cannot be reproduced in an AMIP setup without nudging, a comparison of monthly average data is more appropriate than comparing daily data.

**Figure 10:**
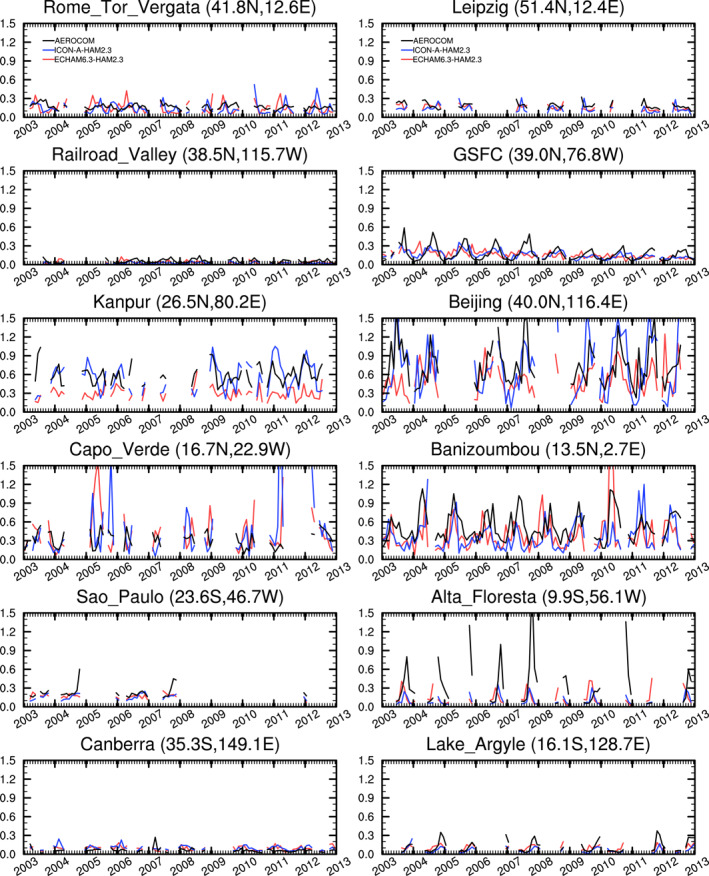
Time series of monthly mean observed and simulated AOT for the years from 2003 to 2012 at selected AERONET stations.

Results for a few selected stations are shown in Figure 10. AERONET observations, ECHAM6.3‐HAM2.3, and ICON‐A‐HAM2.3 all show major differences of the AOT magnitude between the stations. At Alta Floresta, which is located near the southern edge of the Amazon, both models underestimate the seasonal maximum AOT. This area is influenced by biomass burning emissions. Sensitivity studies with ECHAM6.3‐HAM2.3, in which the biomass burning emissions from ACCMIP were replaced by biomass burning emissions from the Global Fire Assimilation System (GFAS; Kaiser et al., 2012), showed smaller AOT biases with respect to MODIS and notably improved the AOTs at Alta Floresta with respect to AERONET (Figures 3 and 4 of Tegen et al., 2019).

**Figure 11:**
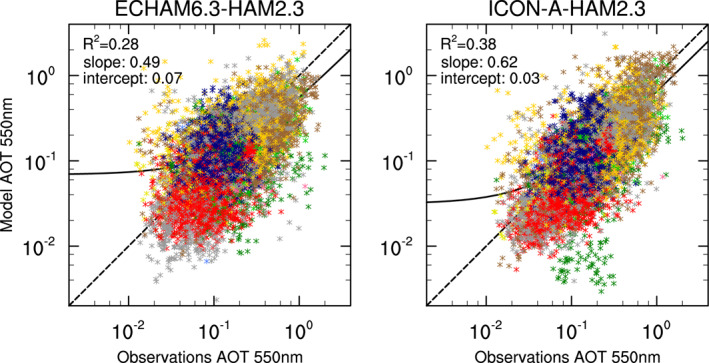
Scatterplots of monthly mean observed versus simulated mean AOT for the years from 2003 to 2012 at 590 AERONET stations shown in Figure 9 but excluding Mauna Loa. The color coding is as in Figure 9 with regions North America (red), South America (dark green), Europe and Western Mediterranean Sea coastal (dark blue), North Africa and Arabian Peninsula (yellow), South Africa (green), East Asia (brown), Siberia (pink), Australia (lemon), oceanic regions (blue), and mixed regions (gray). The black line is the least squares linear fit. R^2^ is the fraction of variance that is explained by this linear fit.

The underestimation of AOT in the northern South American region (green points in the map in Figure 9) is especially pronounced in ICON‐A‐HAM2.3 (green points in the scatterplot in Figure 11), in agreement with the larger bias in this region relative to the MODIS data in Figures 2b and 2c. As indicated in the previous section, this can be attributed to different settings of *δp*_min_. Both models tend to reproduce the annual cycle at the selected stations, although at the Kanpur site, which is located in northern India, the seasonal cycle is stronger in ICON‐A‐HAM2.3 and in better agreement with the observations. In Cape Verde, ECHAM6.3‐HAM2.3 simulates a dust event in the first half of 2005 and ICON‐A‐HAM2.3 simulates a dust event in the second half of 2005 and also again later in the time series. That dust events are out of phase with the observations is expected in model runs without nudging of meteorological data to observations. Because of the relatively large amount of missing data in the observations, it is difficult to assess whether the magnitude of the events is realistic.

On the whole, ICON‐A‐HAM2.3 AOTs show a higher linear correlation to monthly mean AERONET AOTs compared to ECHAM6.3‐HAM2.3 (Figure 11). The squared Pearson correlation coefficient is 0.28 in ECHAM6.3‐HAM2.3 and 0.38 in ICON‐A‐HAM2.3. This higher correlation could at least partially be explained by a higher horizontal resolution in ICON‐A‐HAM2.3 (around 160 km globally in ICON‐A‐HAM vs. 200 km at the equator in ECHAM6.3‐HAM2.3), and partially by higher AOTs over southern and eastern Asia (East Asia region, brown points in Figures 9 and 11).

### 4.5 Sea Salt and BC in the Upper Troposphere Over the Pacific

**Figure 12:**
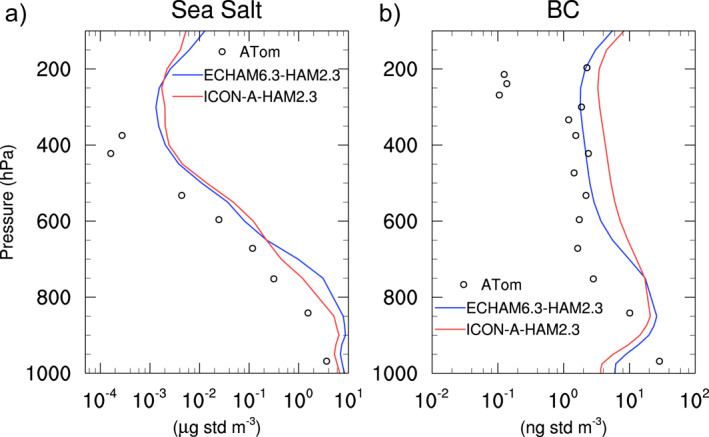
Vertical profiles of horizontally averaged sea salt (a) and BC (b) concentration at standard conditions from the Atmospheric Chemistry, Trace Gases, and Aerosols (ATom) campaign compared to climatological model averages for the region from 170°E to 90°W and 15°S to 10°N for ECHAM6.3‐HAM2.3 and ICON‐A‐HAM2.3.

The concentrations of sea salt and BC aerosol over the Pacific ocean vary strongly between the surface and the upper troposphere (Yu et al., 2019). Sensitivity studies with ECHAM6.3‐HAM2.3 previously yielded a strong dependence of simulated vertical aerosol profiles on wet deposition (Watson‐Parris et al., 2019). Figure 12 compares horizontally averaged observations from the ATom field campaign (Wofsy et al., 2018) to climatological means of simulated sea salt and BC concentrations in the region from 170°E to 90°W and 15°S to 10°N. The sea salt concentration decreases by several orders of magnitude between the lower and the upper troposphere in ICON‐A‐HAM2.3, ECHAM6.3‐HAM2.3 and the ATom data. The sea salt concentration in the lower troposphere is lower in ICON‐A‐HAM2.3 compared to ECHAM6.3‐HAM2.3. The difference of the sea salt concentration in the upper troposphere between ICON‐A‐HAM2.3 and ECHAM6.3‐HAM2.3 is moderate in spite of decreased scavenging factors in convective clouds in ICON‐A‐HAM2.3. This can in part be explained by less sea salt being available for transport to the upper troposphere in ICON‐A‐HAM2.3 (Figure 12a). Less sea salt in the lower troposphere is consistent with the lower AOTs due to decreased *δp*_min_.

The BC concentration in the boundary layer is lower in the climatological mean profiles from the models compared to the ATom data. Especially ICON‐A‐HAM2.3 shows higher concentrations in the upper troposphere compared to the averaged ATom profiles. A comparison of vertical profiles from ICON‐A‐HAM2.3 and ICON‐A‐HAM2.3 RBT, which is identical to ICON‐A‐HAM2.3 except higher scavenging factors (Table 3), shows that decreasing scavenging factors in ICON‐A‐HAM2.3 leads to higher concentrations of sea salt and BC in the upper troposphere in the region from 170°E to 90°W and 15°S to 10°N (Figure S14 in Supporting Information S1), which agree less well with the horizontally averaged observations from the ATom field campaign. On the other hand, the lower tropospheric BC concentrations in Figure S14 in Supporting Information S1 agree better with the ATom data in ICON‐A‐HAM2.3 than in ICON‐A‐HAM2.3 RBT, and reduced scavenging factors also resulted in reduced negative AOT biases in Figure 2.

### 4.6 Global Mean Aerosol Burdens and Lifetimes

Table 5 shows simulated aerosol burdens and lifetimes for sulfate, BC, OC, dust, and sea salt aerosol and provides an overview of the sources and sinks of these aerosol types for ECHAM6.3‐HAM2.3, ICON‐A‐HAM2.3, and for the RBT and NT sensitivity runs.

**Table 5:** Aerosol Budgets Globally Averaged Over 2003–2012

	ECHAM6.3‐ HAM2.3	ICON‐A‐ HAM2.3	ICON‐A‐ HAM2.3 RBT	ICON‐A‐ HAM2.3 NT	AeroCom Textor et al. (2006)
Sulfate					
Burden Tg (S)	0.73	0.76	0.66	0.63	0.67
Production + Emissions	70.5	74.3	73.7	69.6	59.7
Sinks (Tg S yr^−1^)					
Sedimentation	0.69	0.87	0.88	0.89	
Dry deposition	2.00	2.28	2.23	2.18	6.9^a^
Wet deposition	68.1	70.7	70.1	66.5	53.0
Lifetime (d)	3.78	3.74	3.30	3.32	4.1
BC					
Burden (Tg)	0.14	0.15	0.13	0.11	0.24
Sources (Tg yr^−1^)					
Emissions	7.82	7.82	7.82	7.82	
Sinks (Tg yr^−1^)					
Sedimentation	0.02	0.03	0.03	0.03	
Dry deposition	0.70	0.76	0.75	0.75	2.6^a^
Wet deposition	7.17	7.00	7.02	7.06	9.4
Lifetime (d)	6.50	7.00	6.29	5.27	7.1
OC					
Burden (Tg)	1.05	1.02	0.89	0.73	1.7
Sources (Tg yr^−1^)					
Emissions	68.5	68.5	68.5	68.5	
Sinks (Tg yr^−1^)					
Sedimentation	0.18	0.28	0.27	0.26	
Dry deposition	5.03	5.12	5.06	5.10	19.2^a^
Wet deposition	63.7	63.0	63.4	65.2	76.7
Lifetime (d)	5.57	5.43	4.71	3.76	6.5
Dust					
Burden (Tg)	18.7	15.6	15.1	12.4	19.2
Sources (Tg yr^−1^)					
Emissions	1226	932	899	823	
Sinks (Tg yr^−1^)					
Sedimentation	405	347	330	309	
Dry deposition	81.5	49.7	46.0	43.3	1235^a^
Wet deposition	748	533	523	471	607
Lifetime (d)	5.53	6.13	6.14	5.48	4.1
Sea salt					
Burden (Tg)	4.07	3.02	2.64	1.97	6.4
Sources (Tg yr^−1^)					
Emissions	1243	1398	1402	1264	
Sinks (Tg yr^−1^)					
Sedimentation	262	239	233	202	
Dry deposition	100	101	100	84.6	4377^a^
Wet deposition	884	1069	1090	1033	1902
Lifetime (d)	1.19	0.78	0.68	0.54	0.41

*Note.* The budgets are compared to AeroCom multi‐model mean values from Textor et al. (2006) as in Tegen et al. (2019).

^a^ For AeroCom results dry deposition also contains sedimentation fluxes.

The budgets and lifetimes of sulfate, OC, and BC are on the whole fairly similar for ICON‐A‐HAM2.3 and ECHAM6.3‐HAM2.3. Larger differences between ECHAM6.3‐HAM2.3 and ICON‐A‐HAM2.3 are found for dust and sea salt in Table 5. This points to accumulation and coarse mode aerosol as a source of differences in simulated AOTs. Especially, the larger differences for sea salt are consistent with the pronounced difference in AOT bias over the subtropical oceans in ECHAM6.3‐HAM2.3 (Figure 2b) compared to ICON‐A‐HAM2.3 (Figure 2c), which we have discussed above in Section 4.1. For dust, on the other hand, Figure 3l shows some of the largest differences between ICON‐A‐HAM2.3 and ECHAM6.3‐HAM2.3 in regions for which MODIS data in Figure 2a is missing, which limits the role of dust for the biases which are found in the comparison to MODIS data in Section 4.1.

For dust and for sea salt, the emissions are computed instead of prescribed and they depend on wind speed and for dust also on soil wetness and snow cover. Furthermore, for dust emission, a constant scaling factor is used in ICON‐A‐HAM2.3 instead of factors that depend on the region (Tegen et al., 2019) as in ECHAM6.3‐HAM2.3. A smaller dust source in ICON‐A‐HAM2.3 (Table 5) leads to smaller sink terms for dust. The atmospheric lifetime of dust with respect to wet deposition, computed as the burden divided by the wet deposition sink, increases by about 1.5 days in ICON‐A‐HAM2.3. The sedimentation and dry deposition changes are less important for the dust lifetime.

The sea salt emission is increased in ICON‐A‐HAM2.3 by 12% relative to ECHAM6.3‐HAM2.3, which is consistent with higher near‐surface (10m) wind speed in ICON‐A‐HAM2.3 compared to ECHAM6.3‐HAM2.3 over subtropical oceans (Figure S1 in Supporting Information S1) as discussed above, but the lifetime is reduced by 34% due to more efficient wet deposition in ICON‐A‐HAM2.3. More efficient wet deposition of sea salt was attributed specifically to wet deposition due to moist convection in Section 4.1. It is consistent with decreased AOTs due to the change of *δp*_min_ which has been discussed earlier in Section 4.3. The more efficient wet deposition is partly compensated by radiation balance tuning and the decrease in scaveing factors associated with convective cloud, leading to an increase of the sea salt lifetime, but the lifetime of sea salt is still 34% below that in ECHAM6.3‐HAM2.3. The combined effect of radiation balance tuning and decreased scavenging factors in ICON‐A‐HAM2.3 is a reduction of wet deposition of sea salt due to convective cloud from 51.7 ng m^−2^s^−1^ (Figure 7j) to 46.6 ng m^−2^s^−1^ (Figure 4k). Although radiation balance tuning was initially performed disregarding changes in AOT, it affects the budgets notably (Table 5).

### 4.7 Effective Radiative Forcing

We compute an effective radiative forcing due to aerosol‐radiation and aerosol‐cloud interactions (ERFari + aci) of −1.45 W m^−2^ for ICON‐A‐HAM2.3 and of −1.09 W m^−2^ for ECHAM6.3‐HAM2.3. ERFari + aci is computed as the difference of TOA net radiation between a run with 2003–2012 (present‐day) and a run with 1850 (pre‐industrial) anthropogenic aerosol emissions. These estimates are in the range of the 68% confidence interval for the total ERFaer of −1.6 to −0.6 W m^−2^ by Bellouin et al. (2020), which is based on multiple lines of evidence including global model results. The ICON‐A‐HAM2.3 NT model setup yields an ERFari + aci of −1.44 W m^−2^. The ICON‐A‐HAM2.3 DLV setup with *δp*_min_ = 300 hPa as in ECHAM6.3‐HAM2.3 yields an ERFari + aci of −1.48 W m^−2^. An ICON‐A‐HAM2.3 setup in which the autoconversion rate of cloud droplets to form rain in the convective parameterization *α*_c_ = 9.0 10^−4^ s^−1^ as in ECHAM6.3‐HAM2.3 (CPC3 in Table 4) yields an ERFari + aci of −1.51 W m^−2^. Neither of these additional ERFari + aci computations can explain the difference of ERFari + aci between ECHAM6.3‐HAM2.3 and ICON‐A‐HAM2.3. Additional model sensitivity runs, potentially including runs to test hypothesis that are unrelated to parameter tuning, would be required to explain this difference. In addition to parameter settings, for example, different background aerosol concentrations or different changes in dust emissions due to changes in wind speed or differences in (low) clouds, which are unrelated to parameter settings, could potentially help to explain the difference. A more detailed analysis of these issues would be required before designing corresponding sensitivity studies, and it is not clear a priori that a single factor explains most of the difference.

### 4.8 Evaluation of Meteorological Variables

**Figure 13:**
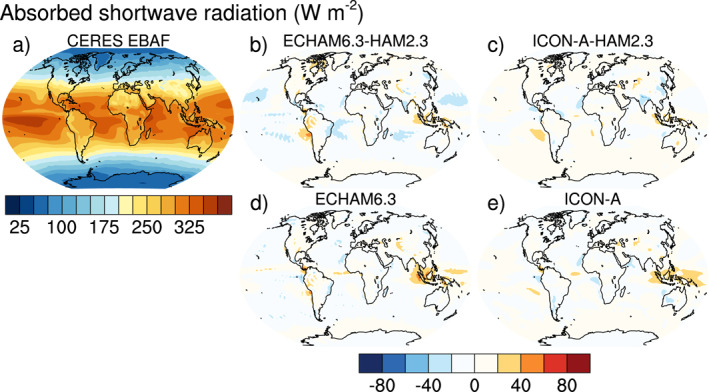
Absorbed shortwave radiation (W m^−2^) computed from the CERES‐EBAF (Loeb et al., 2018) data set for the years 2003–2012 (a) and difference between simulated fields for ECHAM6.3‐HAM2.3, ICON‐A‐HAM2.3, ECHAM6.3, and ICON‐A and CERES‐EBAF data (b)–(e).

**Figure 14:**
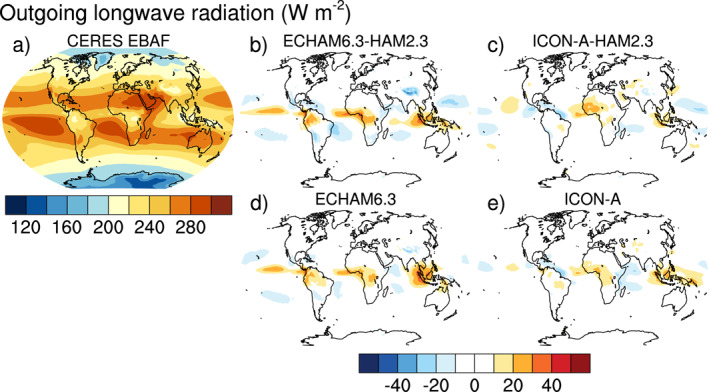
Same as Figure 13 for outgoing longwave radiation.

**Figure 15:**
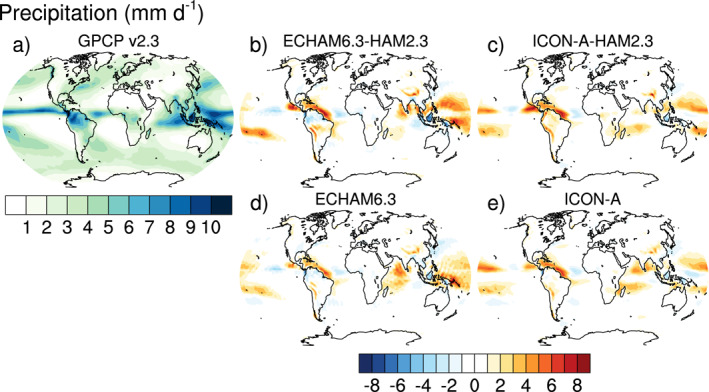
Precipitation (mm d^−1^) from the GPCPv2.3 data set (a). Difference between between simulated fields for ECHAM6.3‐HAM2.3, ICON‐A‐HAM2.3, ECHAM6.3, and ICON‐A and GPCPv2.3 (b)–(e).

Model results for meteorological variables depend, among other factors, such as the model resolution and the choice of parameterization schemes, on the radiation balance tuning choices. The main goal of radiation balance tuning is to reduce the imbalance in the global mean net radiation at the TOA. This is usually achieved via tuning cloud parameters. In order to avoid that the TOA net radiation imbalance is reduced at the cost of introducing large errors in the contributions from individual cloud types, it is instructive to look at separate maps of TOA shortwave and longwave radiation. Figure 13 shows the absorbed shortwave radiation (SWABS) based on the CERES EBAF data set and model biases. A corresponding plot for the outgoing longwave radiation (OLR) is shown in Figure 14. We also compare precipitation maps from the GPCP v2.3 data set to model results in Figure 15. Global mean values of several variables are compared in Table 6.

**Table 6:** Global Mean Short‐Wave Absorption (SWABS), Outgoing Long‐Wave Radiation (OLR), Net Radiation at the Top of the Atmosphere (netradTOA), Short‐Wave Cloud Radiative Effect (SWCRE), Long‐Wave Cloud Radiative Effect (LWCRE), Precipitation (P), Liquid Water Path (LWP), Ice Water Path (IWP), and Aerosol Optical Thickness (AOT) From Models and Observation‐Based Estimates (OBS)

	ECHAM6.3‐HAM2.3	ICON‐A‐HAM2.3	ECHAM6.3	ICON‐A	OBS
SWABS (W m^−2^)	238	243	239	242	241^a^ (240.1 ± 2^b^)
OLR (W m^−2^)	238	242	239	241	240^a^ (239.3 ± 3.3^b^)
netradTOA (W m^−2^)	−0.28	1.23	−0.47	0.81	0.92^a^ (0.5–1.0^c^)
SWCRE (W m^−2^)	−50.1	−44	−46.9	−43.4	−45.3^a^ (−47.5 ± 3^b^)
LWCRE (W m^−2^)	24.4	19.8	24.5	22.7	25.8^a^ (26.7 ± 4^b^)
P (mm d^−1^)	3.00	3.05	2.93	2.98	2.71 ± 0.19^d^
LWP (ocean) (g m^−2^)	70.8	61.3	65.2	51.5	42.9–89.4^e^
IWP (g m^−2^)	14.9	35	29.5	25.9	25 ± 7^f^
AOT	0.13	0.11	‐	‐	0.16^g^

^a^ CERES‐EBAF Ed4.1 for the years 2003–2012 (Loeb et al., 2018).

^b^ For years 2000–2010 from Stephens et al. (2012).

^c^ Range for years 2000–2010 from Trenberth et al. (2014).

^d^ Central value from Adler et al. (2018), range from Adler et al. (2012).

^e^ Neubauer et al. (2019) based on Platnick et al. (2015a); Platnick et al. (2015b); Platnick et al. (2017), ATSR2‐AATSR v2.0 (Stengel et al., 2017; Poulsen et al., 2017), Elsaesser et al. (2017).

^f^ Neubauer et al. (2019) based on Li et al. (2012).

^g^ MODIS collection 6.1 level‐3 data for the years 2003–2012 (Platnick et al., 2015a); (Platnick et al., 2015b).

ECHAM6.3‐HAM2.3 and ICON‐A‐HAM2.3 both show larger biases in SWABS compared to the respective host models for the stratocumulus deck west off the coast of Peru where too little solar radiation is absorbed due to a lack of stratocumulus clouds or a lack of reflection by these clouds in the models. Low biases over the subtropical oceans, indicating an overestimation of reflectivity in ECHAM6.3‐HAM2.3 are reduced in ICON‐A‐HAM2.3. These biases are found mainly in ECHAM6.3‐HAM2.3, but not in ECHAM6.3, pointing at either cloud tunings or scattering by aerosols themselves or aerosol‐induced changes in cloud optical properties as possible contributors. ICON‐A‐HAM2.3 shows fairly small SWABS and OLR biases over the Pacific warm pool. The reduction of the positive OLR bias is consistent with a higher global mean ice water path (IWP) in ICON‐A‐HAM2.3 (Table 6). Note, that the autoconversion rate of cloud water to form rain in convective clouds (*α*_*c*_) has been reduced in ICON‐A‐HAM2.3 compared to ECHAM6.3‐HAM2.3. In spite of this, the long‐wave cloud radiative effect (LWCRE) is lower in ICON‐A‐HAM2.3 than in ECHAM6.3‐HAM2.3. As in many other global models, the global mean precipitation rate (Table 6) is higher in the models than in GPCP observation data. The positive bias is larger in ICON‐A‐HAM2.3 than in the other models.

### 4.9 Combined Multivariable Biases of Meteorological Variables

**Figure 16:**
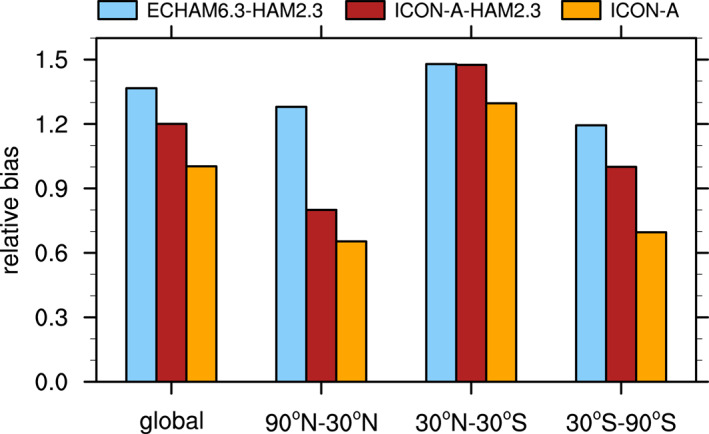
Multivariable biases with respect to observations for the years 2003–2012 normalized by the bias from ECHAM6.3. A relative multivariable bias smaller/larger than 1 indicates a smaller/larger multivariable bias than for ECHAM6.3. The underlying relative biases for individual variables are shown in Figure 17. Information regarding the underlying observation‐based datasets is presented in Section 3.

**Figure 17:**
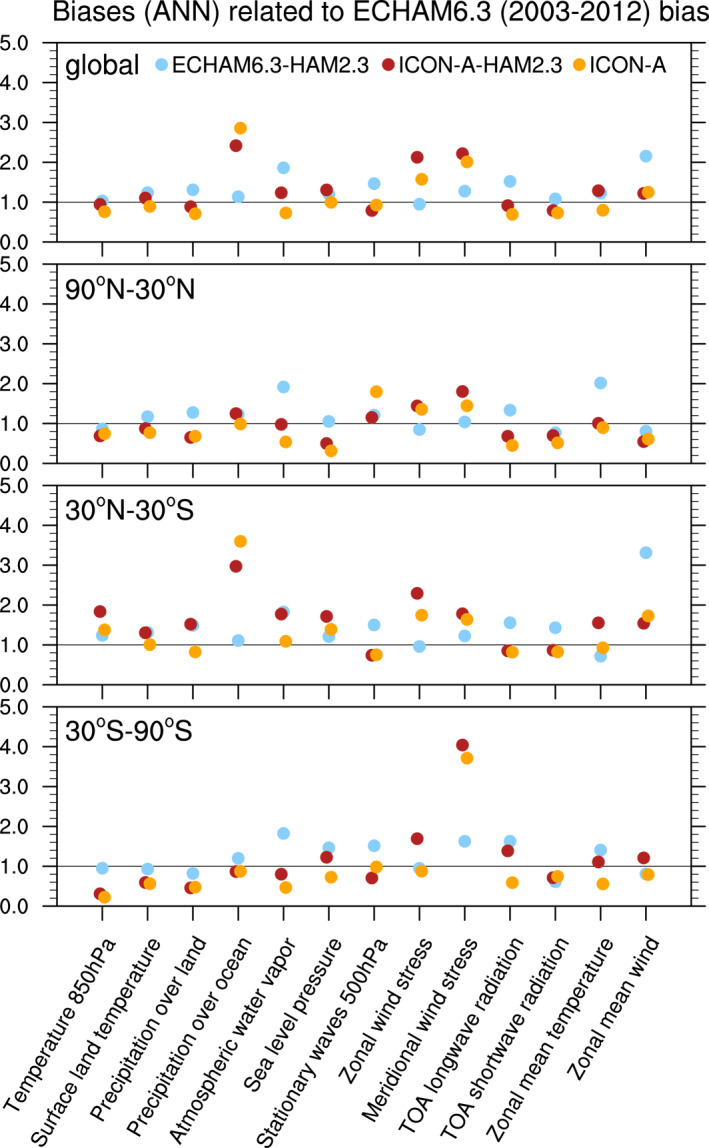
Relative biases as in Figure 16 for individual variables. Zonal mean wind and zonal mean temperature are taken below 10 hPa. The stationary waves bias is based on the 500 hPa geopotential from ERA‐Interim.

For a rough initial assessment of the overall biases of meteorological variables we follow Crueger et al. (2018) motivated by, among others, Reichler and Kim (2008). Figure 16 shows combined multivariable biases with respect to observations for the years 2003–2012 for ECHAM6.3‐HAM2.3, ICON‐A‐HAM2.3, and ICON‐A (icon‐aes‐1.3.00) normalized by the bias from ECHAM6.3. A value of this multivariable relative bias (MVRB) smaller/larger than 1 indicates a smaller/larger multivariable bias than the multivariable bias in ECHAM6.3. The underlying relative biases for individual variables are shown in Figure 17. High relative biases of individual variables can occur in cases in which the bias of ECHAM6.3 is particularly low.

Globally, ICON‐A‐HAM2.3 yields a smaller MVRB than ECHAM6.3‐HAM2.3. This is because in the extra‐tropics (here loosely defined as regions poleward of 30°), ICON‐A‐HAM2.3 shows a smaller MVRB than ECHAM6.3‐HAM2.3 (Figure 16), which apart from a few notable exceptions is reflected by smaller biases in many individual variables (Figure 17). The smaller MVRB in the extratropics in ICON‐A‐HAM2.3 than in ECHAM6.3‐HAM2.3 is consistent with a smaller multivariable bias in the extra‐tropics in ICON‐A than in ECHAM6.3. The MVRB in the tropics (here this refers to the region between 30°N and 30°S) in Figure 16 is of almost identical magnitude for ECHAM6.3‐HAM2.3 and for ICON‐A‐HAM2.3.

Figure 16 also contains information on the increase in multivariable bias between the host models and the HAM‐coupled models. The tropical MVRB in ECHAM6.3‐HAM2.3 and the southern extra tropical MVRB in ICON‐A‐HAM2.3 show particularly large increases relative to the respective host model biases. In both cases several individual variables contribute to this increase. On the whole, the increase of MVRB between the host model and and the corresponding HAM‐coupled model is larger for ECHAM6.3 than for ICON‐A. This may in part be a direct consequence of different radiation balance tuning parameter settings which is largely independent of the associated aerosol changes.

### 4.10 Numerical Noise

**Figure 18:**
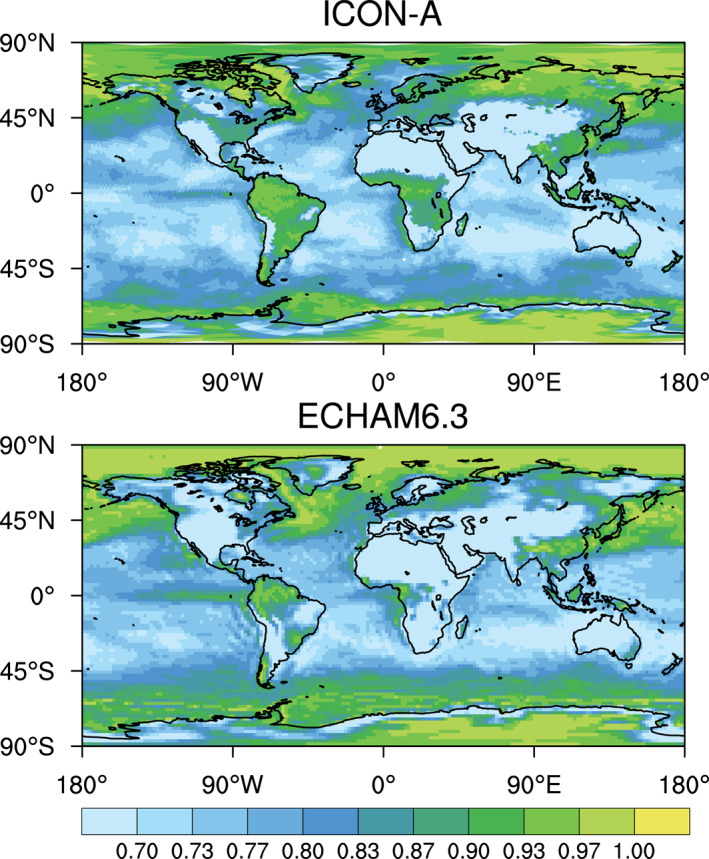
Maps of monthly mean relative humidity in January at the lowest model level from ICON‐A and ECHAM6.3 for illustrating small scale features such as the wavy structure around the Andes in ECHAM6.3. The models are running in AMIP mode (see Section 2.5) and the fields should not be compared directly because these maps do not represent a deterministic forecast for a specific date.

Numerical noise is common in numerical models (Geil & Zeng, 2015), and is not easily addressed without introducing artificial diffusion. In this section we briefly discuss spurious small scale features in the simulated variables originating from the different host model dynamical cores. Figure 18 compares monthly mean relative humidity (RH) in the lowest model level from ICON‐A and ECHAM6.3 at the grid resolutions described in Section 2.5. The ECHAM6.3 RH shows a wave‐like structure in the vicinity of steep orographic features for example, in the Eastern Pacific stratocumulus region, which is readily apparent in Stevens et al. (2013) and also in the ECHAM6.3‐HAM2.3 AOTs in Figure 2b. Reminiscent of gravity waves, this pattern is a numerical artifact.

The ICON‐A RH, on the other hand, displays signs of numerical noise between adjacent triangles, best visible orthogonal to strong gradients such as south of the Sahara. This small‐scale pattern appears to be compatible with the truncation error of the divergence operator switching sign between adjacent triangles as discussed by Wan et al. (2013). The pattern is smoothed out in previous plots where the ICON‐A output data were interpolated to the Gaussian grid.

In the case of RH, numerical noise could in principle influence aerosol growth through water uptake and droplet activation (scavenging), and may also affect the comparison to observations as evidenced by the wavy structure over the southern subtropical Pacific in Figure 2b. Such a wavy structure is also found in cloud fields and in precipitation in standard ECHAM6 (see e.g., Figure 5 Stevens et al. (2013)). This points to aerosol growth through water uptake and droplet activation as a source of the similar wavy pattern in Figure 2. However, compared to the parameter settings in the physics parameterizations, numerical noise most likely plays a negligible role for the overall quality of the results as evidenced by the much larger differences between Figures 2e and 2f compared to the amplitude of the pattern in Figure 2b.

## 5 Conclusions and Discussion

We introduced the ICON‐A‐HAM2.3 atmosphere‐aerosol model and investigated the influence of using a different host model on simulated AOTs. A positive AOT bias over subtropical oceans present in ECHAM6.3‐HAM2.3 has been reduced in ICON‐A‐HAM2.3. Lower AOTs over subtropical oceans and over parts of the tropical oceans in the main subsidence regions outside the Intertropical Convergence Zone and South‐Pacific Convergence Zone in ICON‐A‐HAM2.3 result from increased wet deposition of sea salt aerosol associated with moist convection. This increased wet deposition over subtropical oceans in ICON‐A‐HAM2.3 compared to ECHAM6.3‐HAM2.3 is attributed to a different default setting of a parameter in the moist convection parameterization of the host models. In ECHAM6.3‐HAM2.3, the minimum pressure difference between cloud top and cloud base before the moist convection parameterization starts to produce rain is set to 300 hPa. The lack of such a requirement in ICON‐A‐HAM2.3 implies that moist convection can start to precipitate and thus start to remove hydrophilic aerosol without growing into deep convection.

This finding demonstrates that simulated AOTs can be very sensitive to parameter settings in the host model. Therefore, evaluating the effect of changing such parameters in the host model can be paramount to understanding differences between AOT simulations and also contribute to identifying potential sources of longstanding biases.

Our study also addresses the broader issue of bias compensation. In this particular study, reducing a positive AOT bias over subtropical oceans present in ECHAM6.3‐HAM2.3 leads to a larger bias of the global mean AOT in ICON‐A‐HAM2.3. This is because negative AOT biases over other regions including the tropical rain forests, which are common to ECHAM6.3‐HAM2.3 and to ICON‐A‐HAM2.3, are no longer compensated in the global mean.

Taking the year 1850 as pre‐industrial baseline, we computed an effective radiative forcing due to aerosol‐radiation and aerosol‐cloud interactions (ERFari + aci) of −1.45 W m^−2^ with ICON‐A‐HAM2.3 for the years 2003–2012, compared to −1.09 W m^−2^ with ECHAM6.3‐HAM2.3. Additional model sensitivity runs are required to explain this difference.

Because coupling an aerosol module and a two‐moment microphysics parameterization to a host model affects meteorology, we compared simulations in ECHAM6.3‐HAM2.3, ICON‐A‐HAM2.3, and in the respective host models to selected observations. A multivariable bias combining several meteorological variables into one number is smaller for ICON‐A‐HAM2.3 than for ECHAM6.3‐HAM2.3 due to smaller biases poleward of 30°. This smaller multivariable bias in the extratropics in ICON‐A‐HAM2.3 compared to ECHAM6.3‐HAM2.3 is consistent with a smaller multivariable bias in the extra‐tropics in ICON‐A compared to ECHAM6.3. The multivariable bias between 30°N and 30°S is almost identical in ICON‐A‐HAM2.3 and in ECHAM6.3‐HAM2.3, in spite of a larger “inherited” bias from the default ICON‐A configuration compared to ECHAM6.3. While HAM‐coupling increases the multivariable bias, this increase is smaller for ICON‐A‐HAM2.3 than for ECHAM6.3‐HAM2.3. This is likely caused by different radiation balance tuning parameter settings.

## Supporting information

Supporting Information S1

## Data Availability

CERES‐EBAF data were obtained from the NASA Langley Research Center Atmospheric Science Data Center https://doi.org/10.5067/TERRA-AQUA/CERES/EBAF_L3B.004.1. GPCP data was provided by the NOAA/OAR/ESRL Physical Sciences Laboratory, Boulder, Colorado, USA, from their Web site at https://psl.noaa.gov/data/gridded/data.gpcp.html. ERA‐interim data sets are available from ECMWF (http://apps.ecmwf.int/datasets/data/interim-full-daily/). We thank the AERONET PIs and Co‐PIs and their staff for establishing and maintaining the almost 600 sites from which observations were used in this investigation.
