# Supplementary material for: The Global Atmosphere‐aerosol Model ICON‐A‐HAM2.3–Initial Model Evaluation and Effects of Radiation Balance Tuning on Aerosol Optical Thickness
Source: J Adv Model Earth Syst. 2022 Apr 2;14(4):e2021MS002699. doi: 10.1029/2021MS002699 (PMC9285428; doi:10.1029/2021MS002699)
Supplement: Supplementary file 1 — Supporting Information S1 [file JAME-14-0-s001.pdf]

Supporting Information for ”The global atmosphere-aerosol model ICON-A-HAM2.3 – Initial model evaluation and effects of radiation balance tuning on aerosol optical thickness”

M. Salzmann[1], S. Ferrachat[2], C. Tully[2], S. Münch[2], D. Watson-Parris[3], D. Neubauer[2], C. Siegenthaler-Le Drian[4], S. Rast[5], B. Heinold[6], T. Crueger[5], R. Brokopf[5], J. Mülmenstädt[1,7], J. Quaas[1], H. Wan[8], K. Zhang[8], U. Lohmann[2], P. Stier[3], I. Tegen[6]

[1]Institute for Meteorology, Universität Leipzig, Leipzig, Germany

[2]Institute of Atmospheric and Climate Science, ETH Zürich, Zürich, Switzerland

[3]Atmospheric, Oceanic and Planetary Physics, Department of Physics, University of Oxford, Oxford, UK

[4]Center for Climate Systems Modeling, ETH Zürich, Zürich, Switzerland

[5]Max Planck Institute for Meteorology, Hamburg, Germany

[6]Leibniz Institute for Tropospheric Research, Leipzig, Germany

[7]Now at: Pacific Northwest National Laboratory, Richland, WA, USA

[8]Pacific Northwest National Laboratory, Richland, WA, USA

Contents of this file

1. Figures S1 to S14

Introduction This file contains additional figures mostly comparing meteorological and aerosol results from ECHAM6.3-HAM2.3, ICON-A-HAM2.3 and various sensitivity runs.

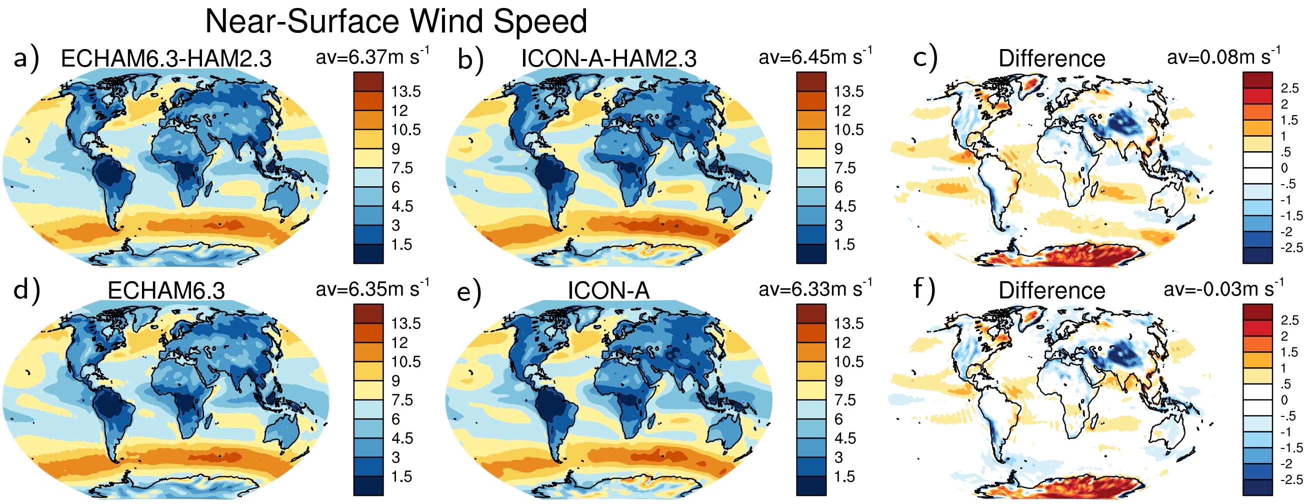

Figure S1. Near-surface (10m) wind speed for the years 2003 to 2012 from ECHAM6.3-HAM2.3 **(a)**, ICON-A-HAM2.3 **(b)**, ECHAM6.3 **(d)**, ICON-A **(e)**. Differences between ICON-A-HAM2.3 and ECHAM6.3-HAM2.3 **(c)** and difference between ICON-A and ECHAM6.3 **(f)** are also shown.

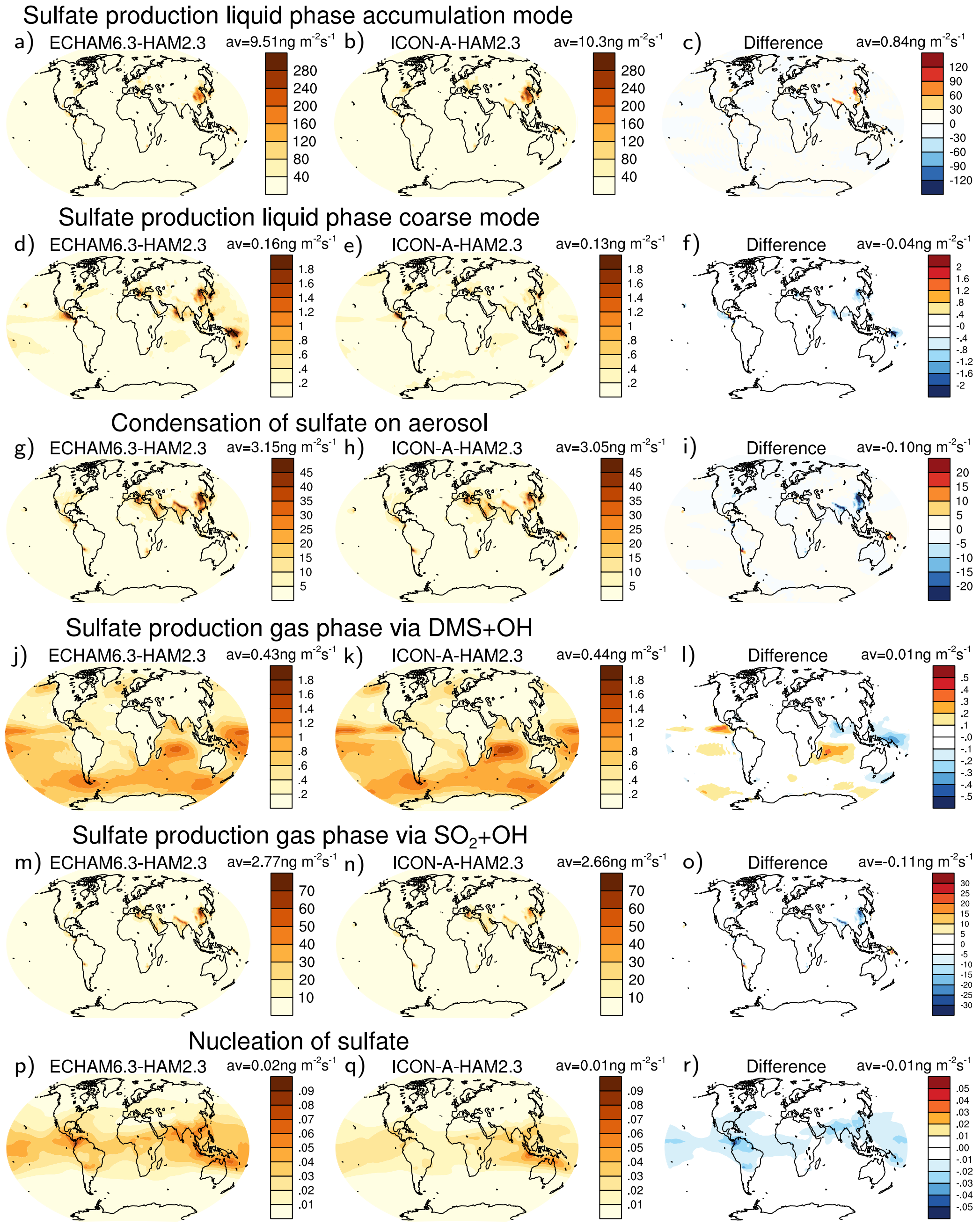

Figure S2. Sulfate production liquid phase accumulation mode **(a-c)**, sulfate production liquid phase coarse mode **(d-f)**, condensation of sulfate on aerosol **(d-f)**, sulfate production gas phase via DMS+OH **(j-l)**, Sulfate production gas phase via SO_2+OH **(m-o)** and nucleation of sulfate **(p-r)** for the years 2003 to 2012 from ECHAM6.3-HAM2.3 (left column) and ICON-A-HAM2.3 (middle) and the difference between ICON-A-HAM2.3 and ECHAM6.3-HAM2.3 (right column).

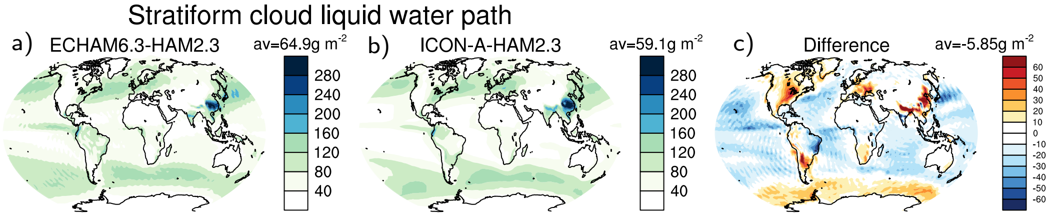

Figure S3. Stratiform cloud liquid water path for the years 2003 to 2012 from ECHAM6.3-HAM2.3 (left column) and ICON-A-HAM2.3 (middle) and the difference between ICON-A-HAM2.3 and ECHAM6.3-HAM2.3 (right column).

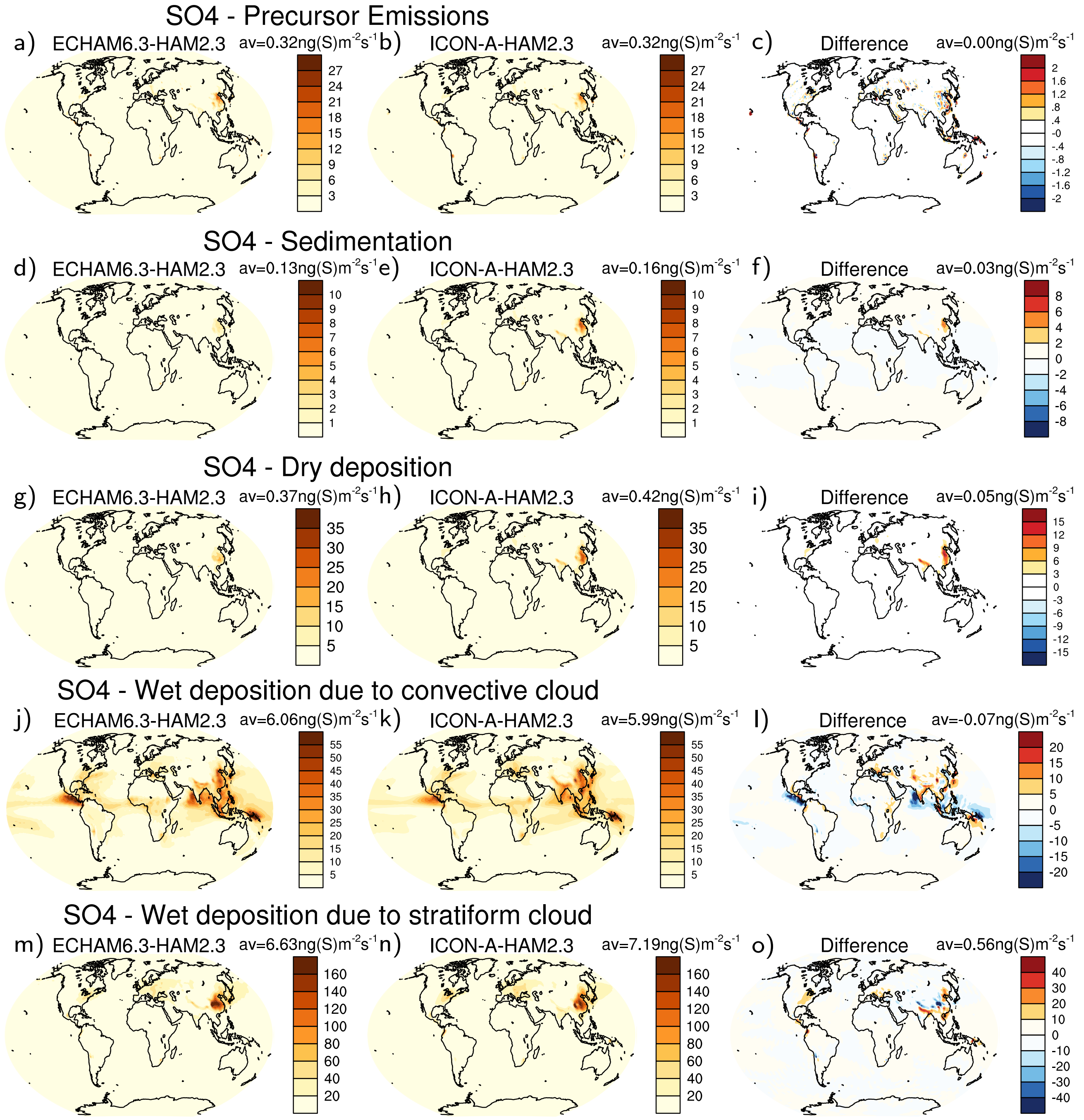

Figure S4. Sulfate aerosol emission **(a-c)**, sedimentation **(d-f)**, dry deposition **(d-f)**, wet deposition due to convective cloud **(j-l)**, and wet deposition due to stratiform cloud **(m-o)** fluxes for the years 2003 to 2012 from ECHAM6.3-HAM2.3 (left column) and ICON-A-HAM2.3 (middle) and the difference between ICON-A-HAM2.3 and ECHAM6.3-HAM2.3 (right column).

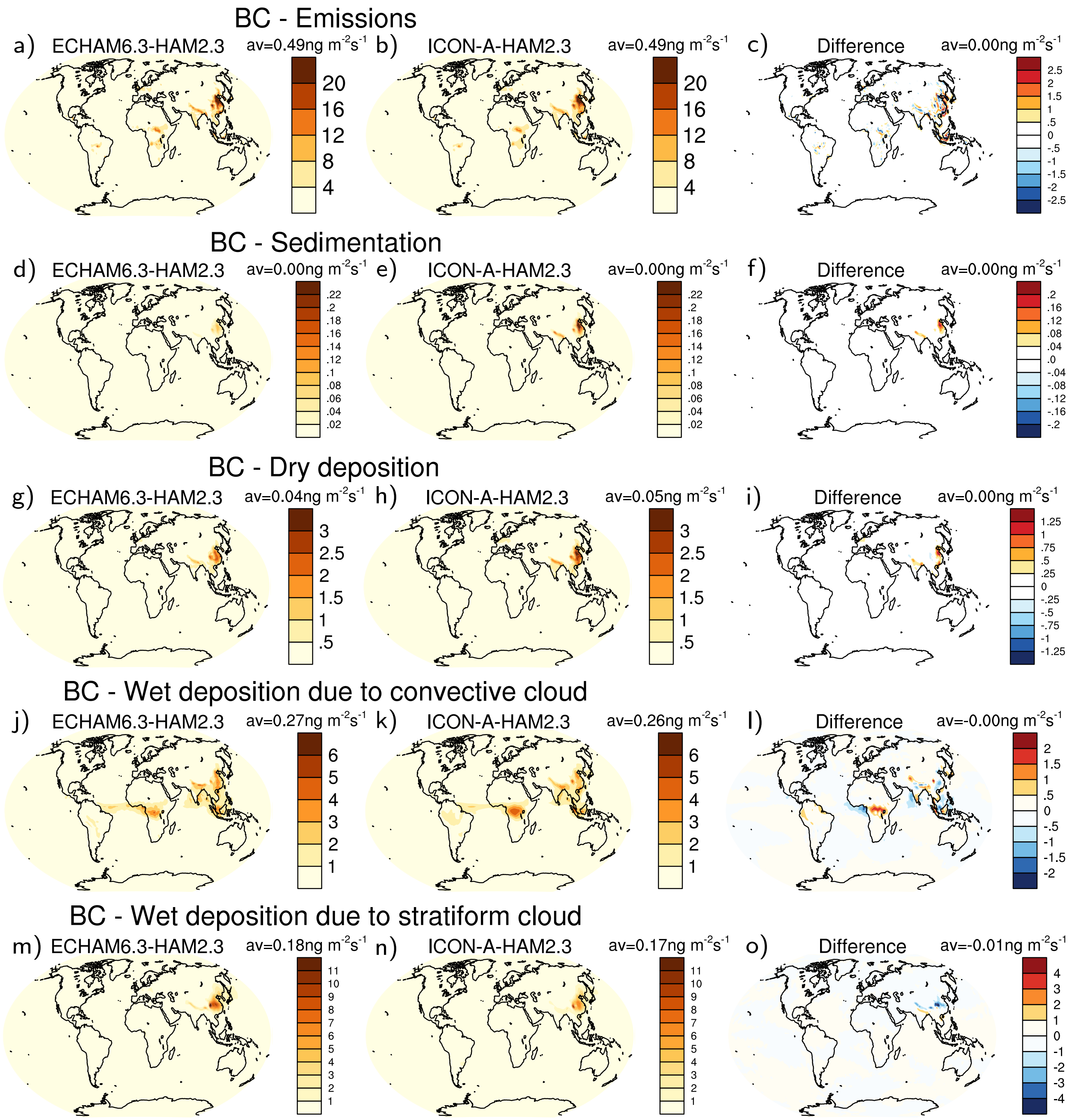

Figure S5. As Figure S4 but for black carbon aerosol.

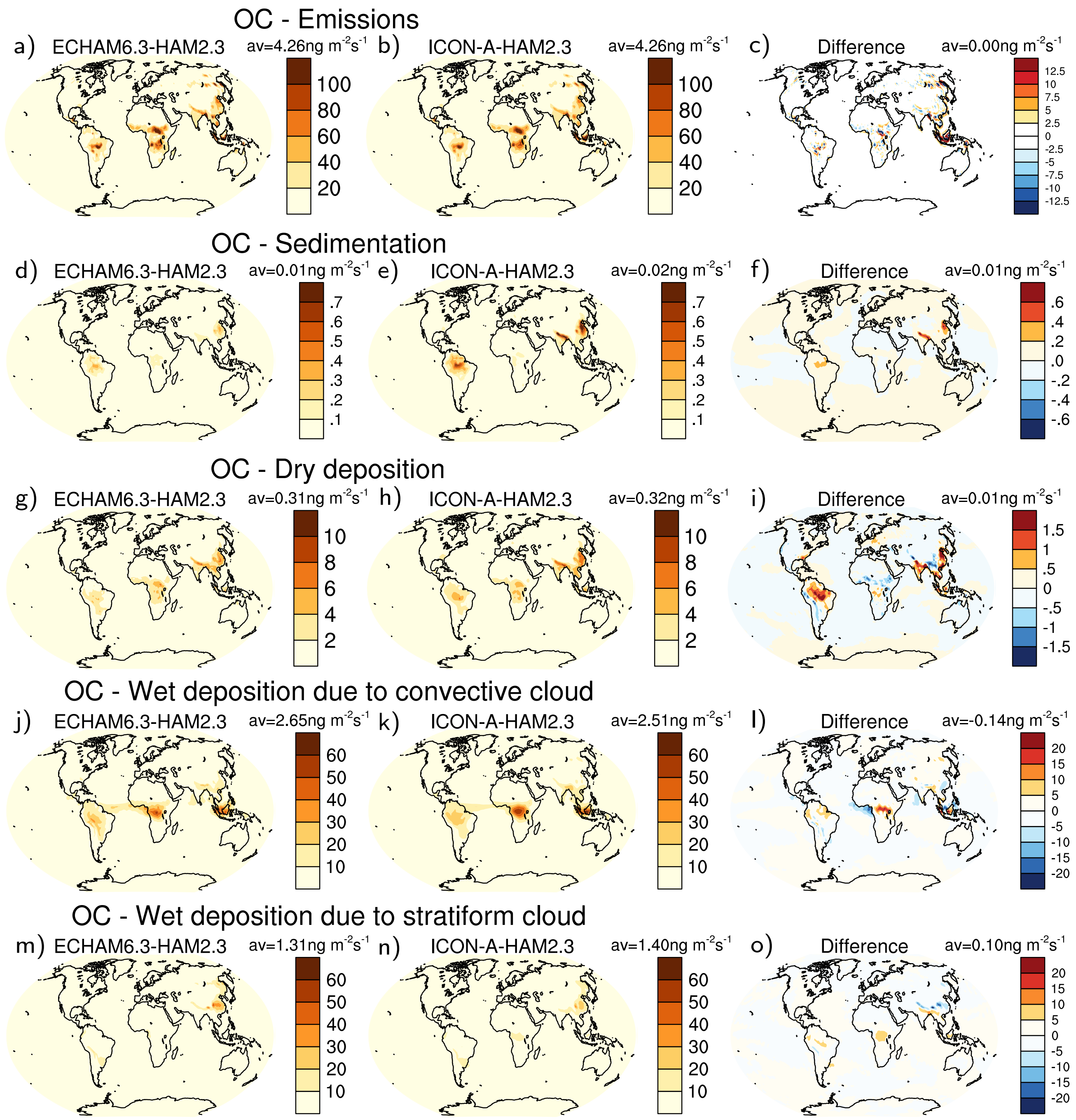

Figure S6. As Figure S4 but for organic carbon aerosol.

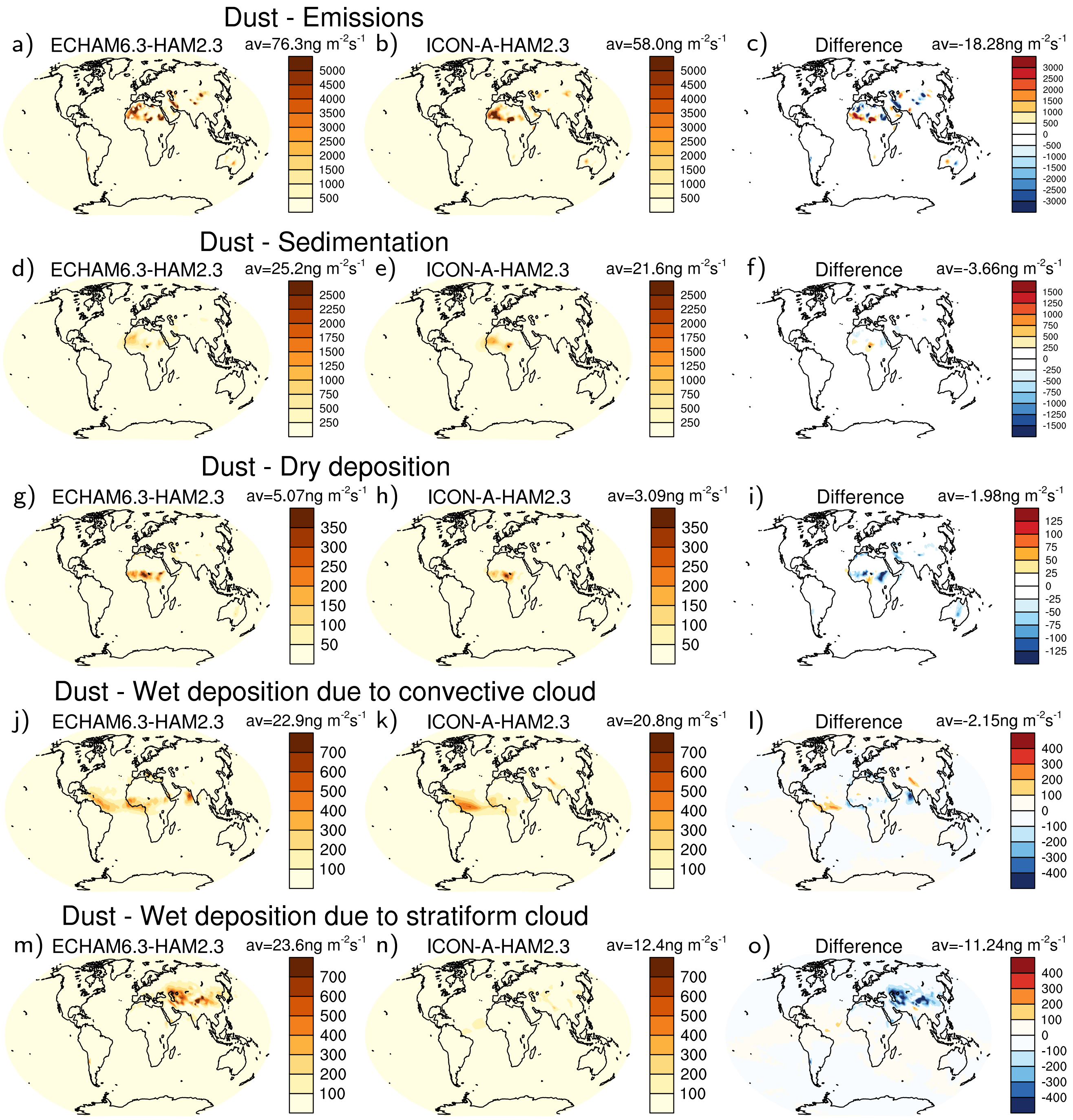

Figure S7. As Figure S4 but for dust aerosol.

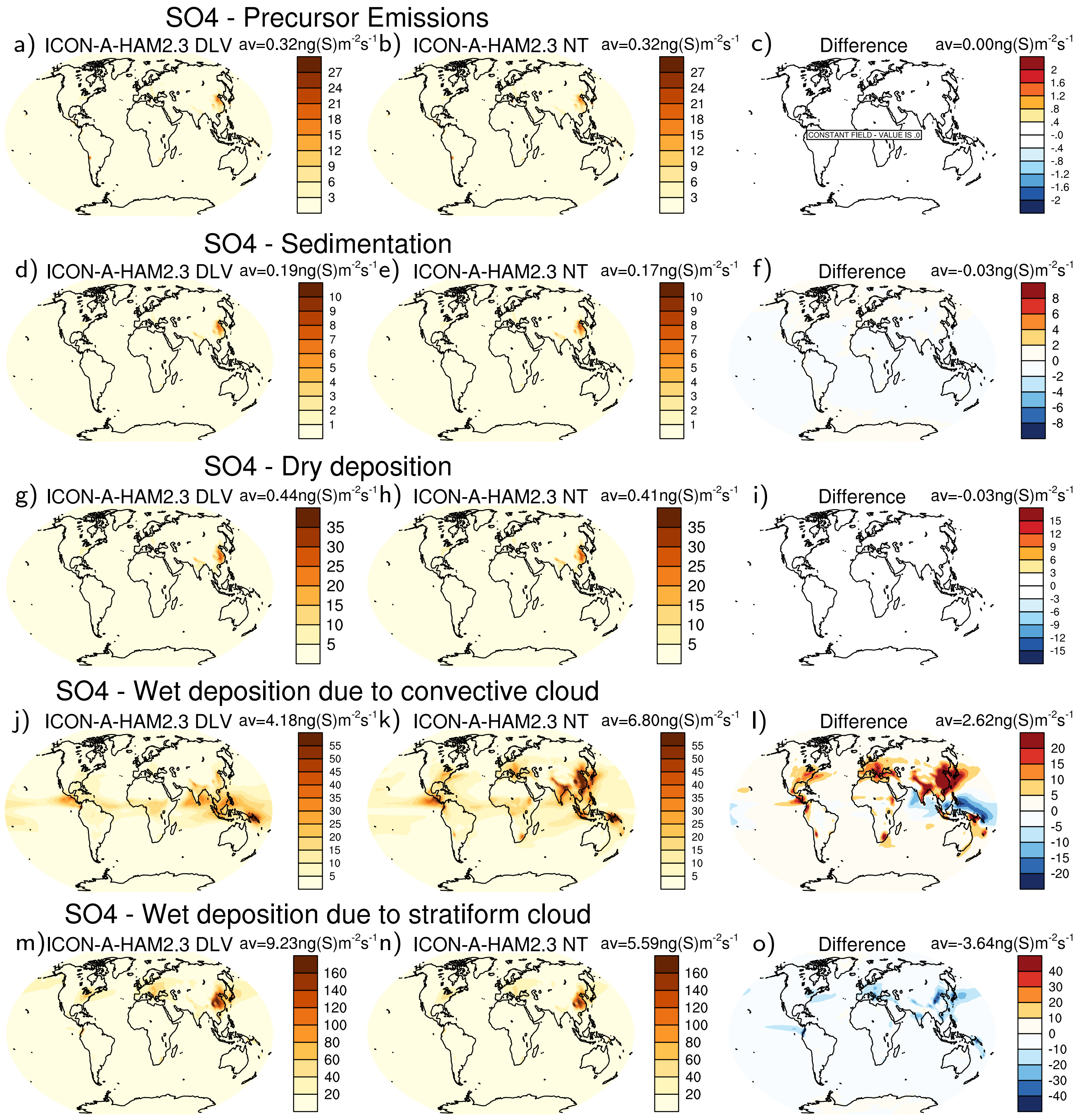

Figure S8. As Figure S4 but for the ICON-A-HAM2.3 DLV sensitivity run (left column) and the ICON-A-HAM2.3 NT sensitivity run (middle) and the difference between the ICON-A-HAM2.3 NT and the ICON-A-HAM2.3 DLV run.

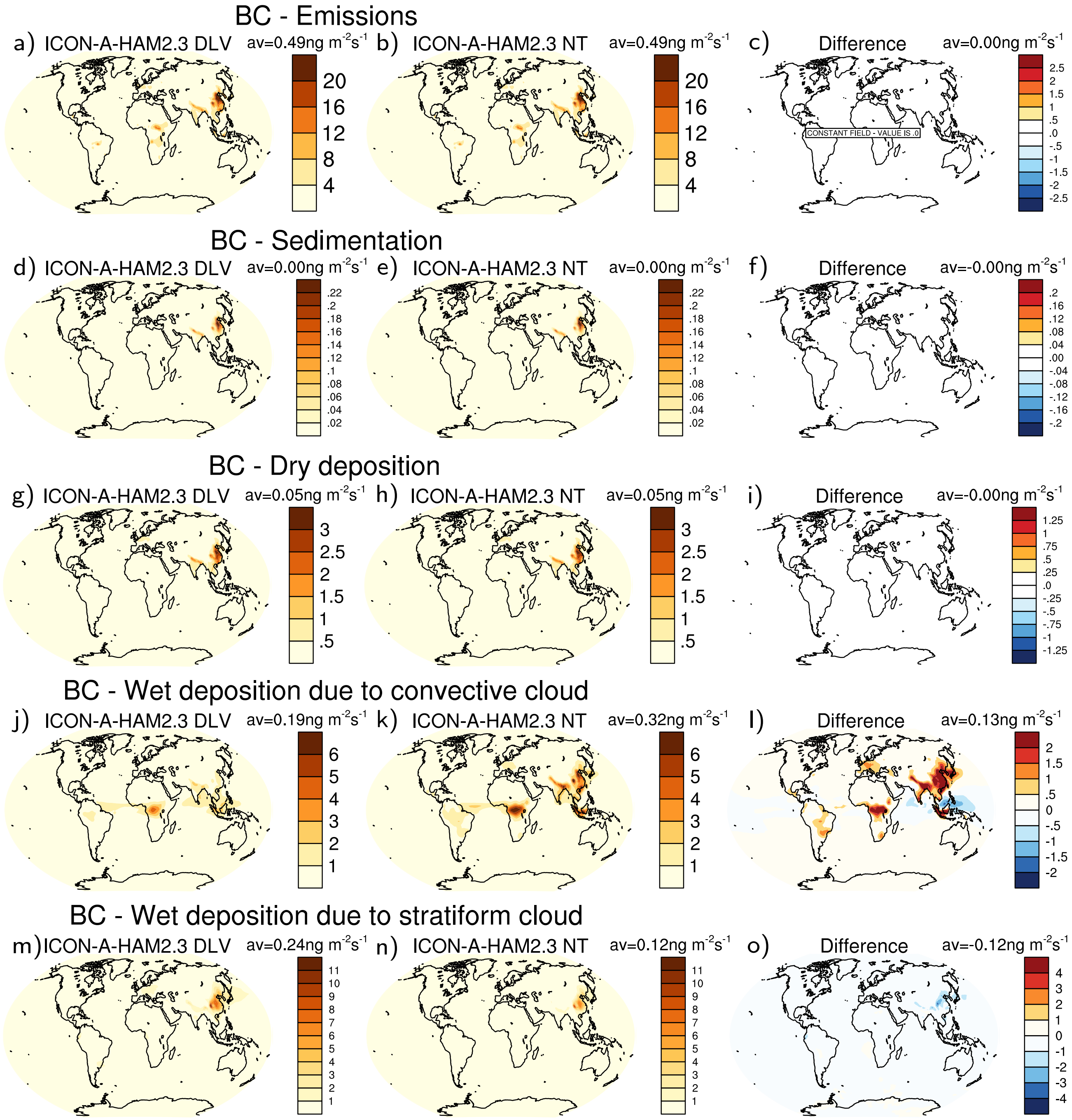

Figure S9. As Figure S8 but for black carbon aerosol.

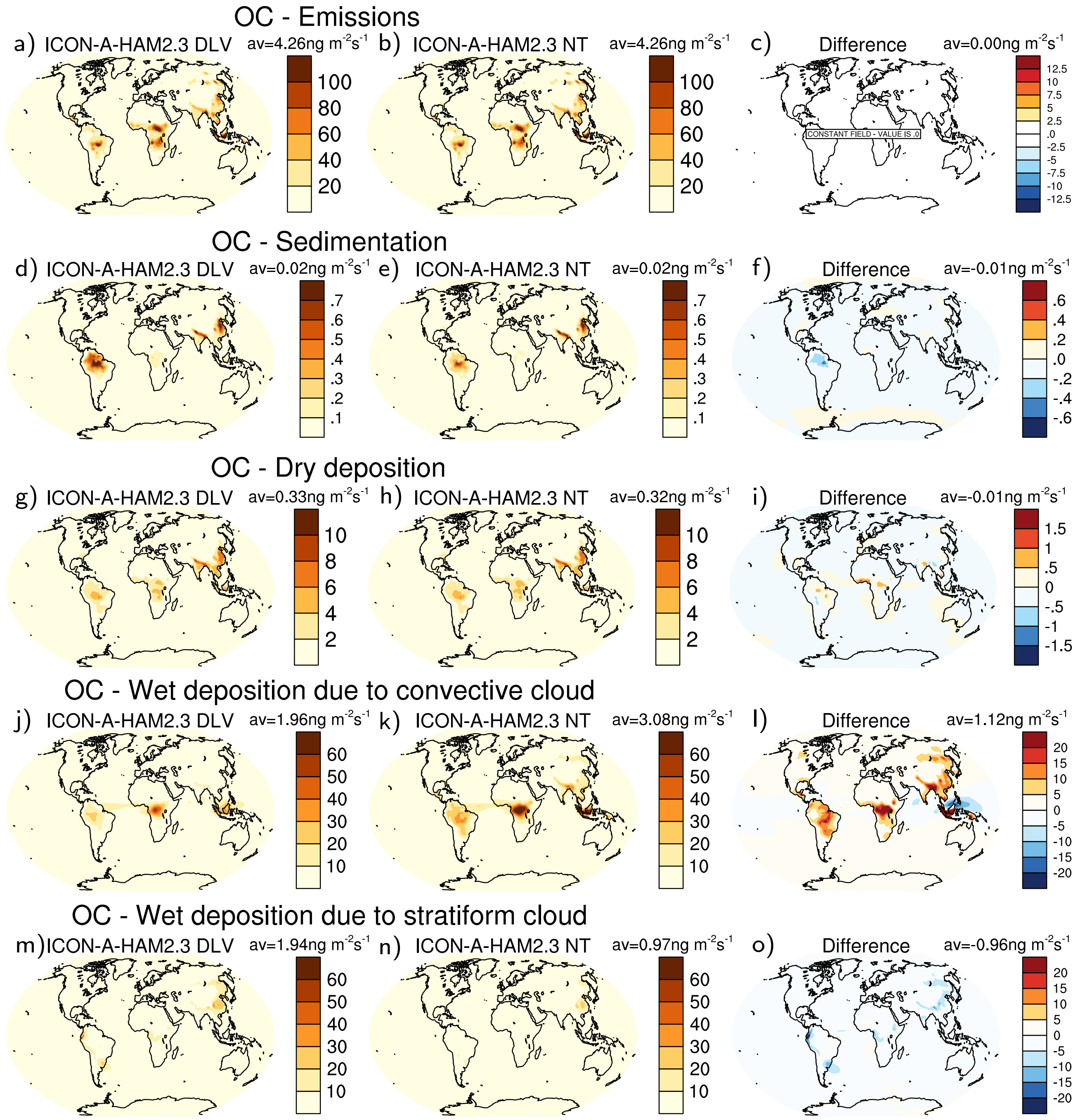

Figure S10. As Figure S8 but for organic carbon aerosol.

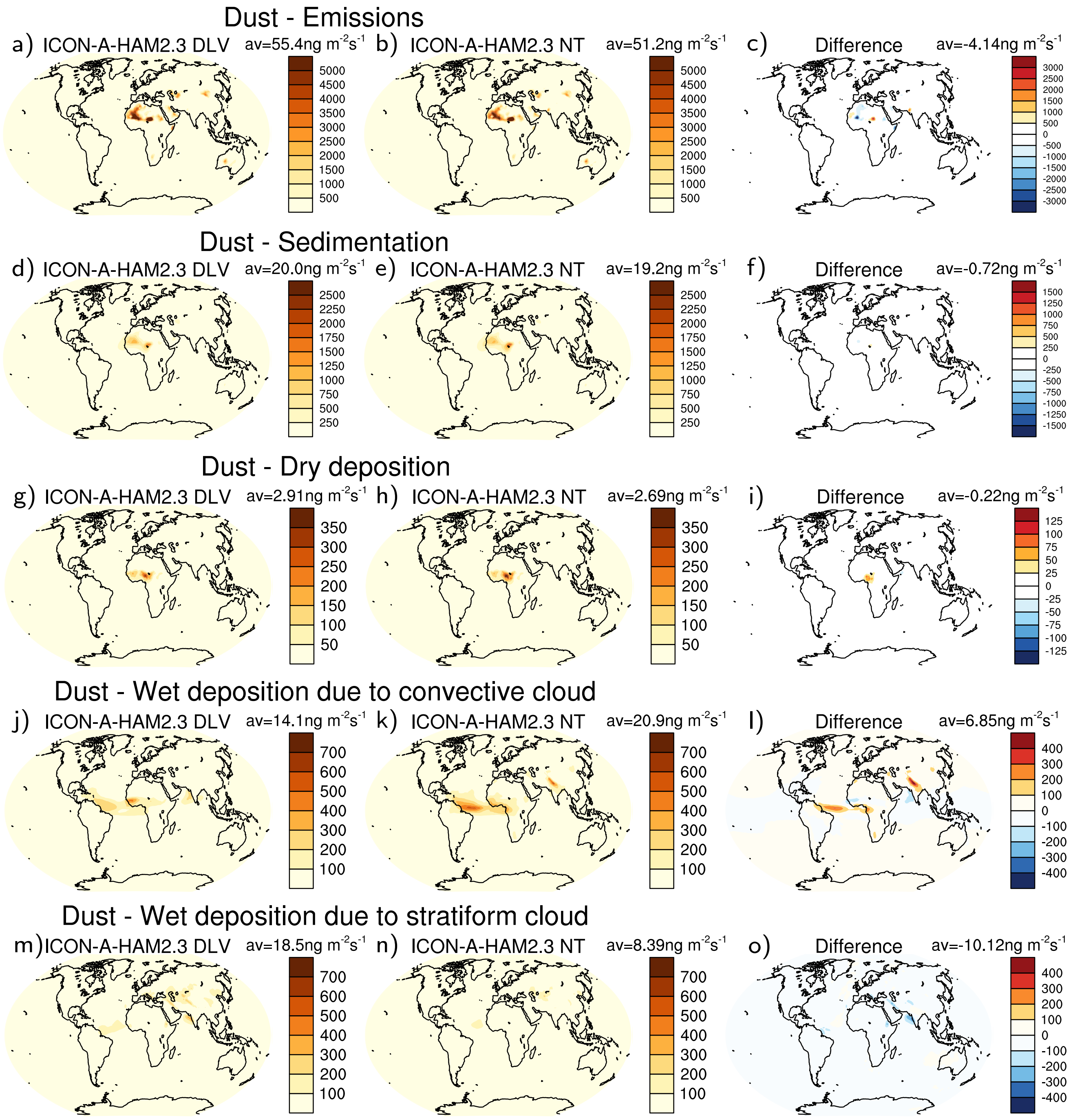

Figure S11. As Figure S8 but for dust aerosol.

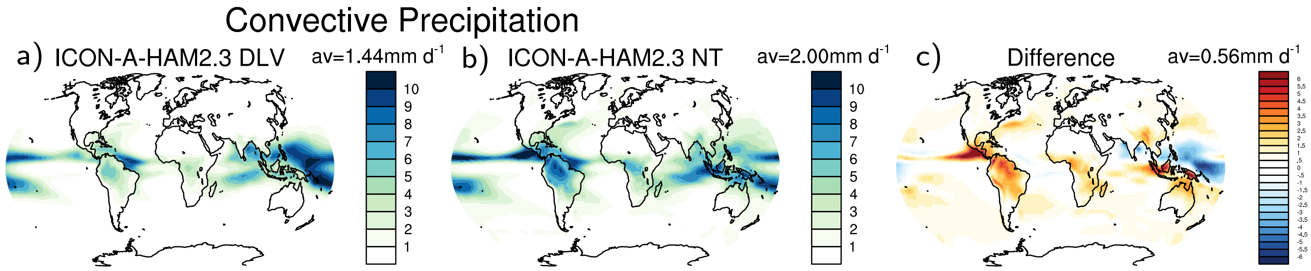

Figure S12. Convective precipitation for the years 2003 to 2012 from the ICON-A-HAM2.3 DLV sensitivity run (left column) and the ICON-A-HAM2.3 NT sensitivity run (middle) and the difference between the ICON-A-HAM2.3 NT and the ICON-A-HAM2.3 DLV run (right column).

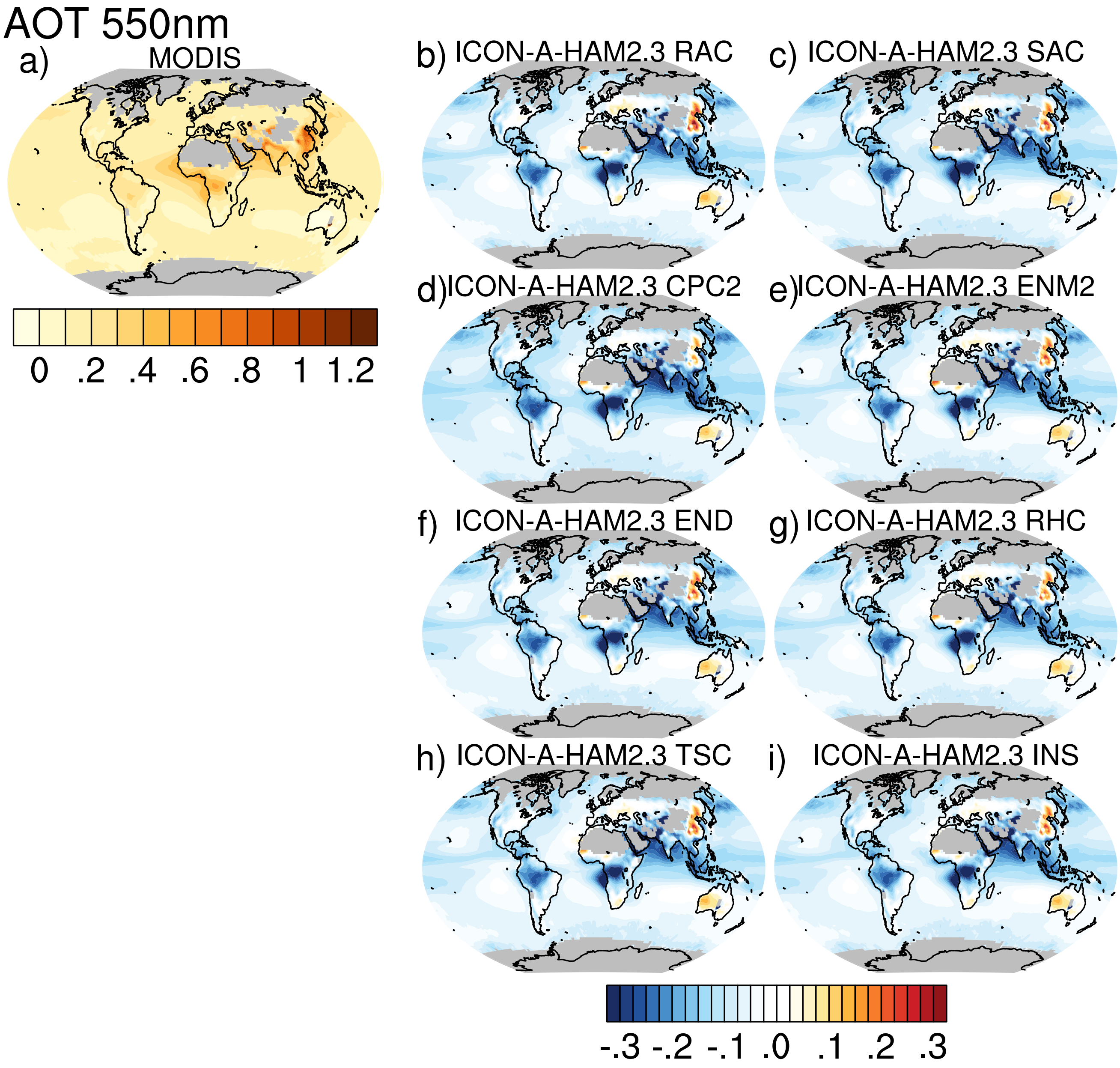

Figure S13. Aerosol optical thickness (AOT) at 550 nm retrieved from MODIS measurements on board the Aqua satellite for the years 2003 to 2012 **(a)**. Difference between model simulated AOT and and MODIS AOT for various sensitivity experiments **(b–i)**

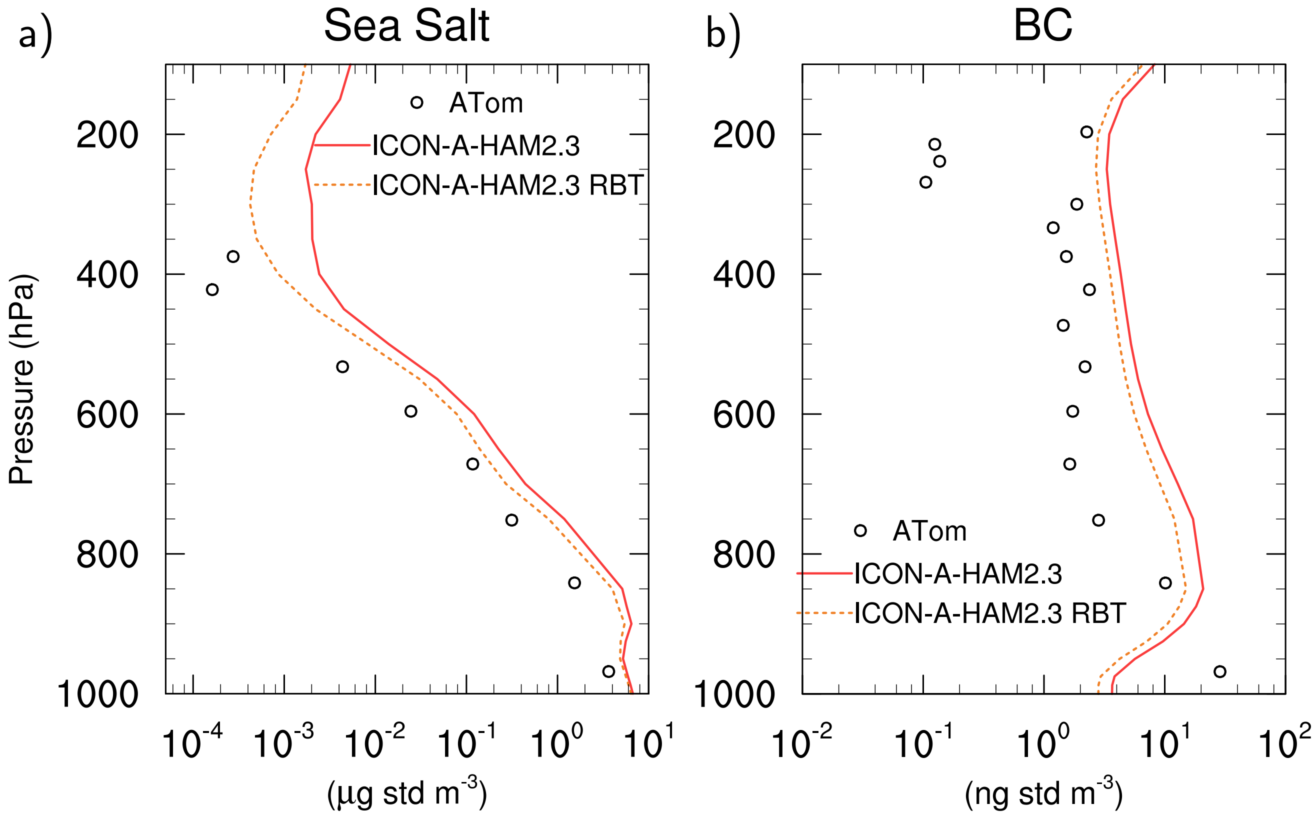

Figure S14. Vertical profiles of horizontally averaged sea salt **(a)** and BC **(b)** concentration at standard conditions from the Atmospheric Chemistry, Trace Gases, and Aerosols (ATom) campaign compared to climatological model averages for the region from 170°E to 90°W and 15°S to 10°N for and ICON-A-HAM2.3 and ICON-A-HAM2.3.
